# Sex-linked helicases DDX3X and DDX3Y regulate G-quadruplex-associated stress in neurons

**DOI:** 10.1038/s41419-026-08971-z

**Published:** 2026-06-18

**Authors:** Rocio Diaz Escarcega, Vijay Kumar M. J., Ashlee Arizmendez, Chunfeng Tan, Akihiko Urayama, Sean P. Marrelli, Rodrigo Morales, Jeffrey S. Wefel, Chunchao Zhang, Louise D. McCullough, Nayun Kim, David Monchaud, Sung Yun Jung, Andrey S. Tsvetkov

**Affiliations:** 1 Department of Neurology, McGovern Medical School, The University of Texas Health Science Center at Houston, Houston, TX USA; 2 The BRAINS Research Laboratory, the Department of Neurology, University of Texas McGovern Medical School, Houston, TX USA; 3 Centro Integrativo de Biologia y Quimica Aplicada (CIBQA), Universidad Bernardo O’Higgins, Santiago, Chile; 4 Department of Neuro-Oncology, The University of Texas MD Anderson Cancer Center, Houston, TX USA; 5 Department of Radiation Oncology, The University of Texas MD Anderson Cancer Center, Houston, TX USA; 6 Department of Otorhinolaryngology, Head and Neck Surgery, The University of Maryland School of Medicine, Baltimore, MD USA; 7 The University of Texas Graduate School of Biomedical Sciences, Houston, TX USA; 8 Division of Pharmacology and Toxicology, College of Pharmacy, The University of Texas at Austin, Austin, TX USA; 9 Institut De Chimie Moléculaire de l’Université de Bourgogne (ICMUB), Université Bourgogne Europe (UBE), Dijon, France; 10 Department of Biochemistry and Molecular Biology, Baylor College of Medicine, Houston, TX USA

**Keywords:** Cellular neuroscience, Alzheimer's disease

## Abstract

G-quadruplexes (G4s) are four-stranded nucleic acid structures that regulate virtually all nucleic acid-dependent cellular processes. At present, most functional studies involving G4s have focused on cancer cells. This study investigated how neurons respond to genotoxic stress induced by quarfloxin (CX-3543), a small molecule that stabilizes G4s. We found that quarfloxin treatment induced DNA damage in neurons, with double-strand breaks enriched in the nucleolus. Proteomic analysis revealed that quarfloxin promoted substantial protein changes, affecting networks associated with Alzheimer’s, Parkinson’s, and Huntington’s diseases, and amyotrophic lateral sclerosis. Among the affected proteins, the G4 helicase DDX3X, encoded on the X chromosome, was upregulated, prompting further investigation of DDX3X and its Y-linked homolog DDX3Y in female and male neurons, respectively. RNA sequencing identified DDX3X- and DDX3Y-regulated gene networks involved in DNA damage responses, inflammation, cell cycle regulation, and stress-associated pathways, with notable sex-dependent differences. In human brain tissue, DDX3X expression and nuclear enrichment were increased in neurons from older females compared to younger individuals, with further elevation observed in Alzheimer’s disease. Taken together, these findings identify DDX3X and DDX3Y as modulators of neuronal stress responses downstream of G4 stabilization and indicate that their induction is accompanied by activation of DNA damage response genes, as well as cell cycle- and inflammation-associated pathways, suggesting that sustained activation of these pathways may disrupt neuronal homeostasis. Our study provides insight into G4-dependent stress mechanisms in neurons and highlights sex-linked pathways that may contribute to brain aging and neurodegenerative disease vulnerability.

## Introduction

Single-stranded guanine (G)-rich sequences can fold into thermodynamically stable four-stranded structures known as G-quadruplexes (G4s), which can form in both DNA (G4-DNA) and RNA (G4-RNA) [1, 2]. G4-DNA has been extensively studied for its regulatory roles in nearly all DNA-dependent cellular processes, including transcription, replication, and telomere maintenance [3, 4]. G4-RNA is increasingly recognized as a regulator of post-transcriptional processes such as mRNA splicing, translation, and stability [5, 6]. G4-DNA-forming sequences (QFS) are abundant in the human genome [7], comprising approximately 1% of the telomere-to-telomere (T2T) genome [8, 9], and are significantly enriched in gene promoters, enhancers, and near replication origins [10, 11]. G4-DNA structures are also found in mitochondrial DNA and are conserved across species, underscoring their functional importance [9, 10, 12]. Mechanistically, G4-DNA tends to form at transcriptionally active sites that are free of nucleosomes [13, 14], where they may facilitate chromatin opening [15–17]. Their broader role in chromatin remodeling is an area of active investigation. Given their regulatory potential, G4 formation is tightly controlled by the cell through a specialized set of proteins, including G4-binding proteins and G4 helicases that resolve them [18–22]. The coordinated activity of these proteins is crucial, and disruptions to this balance can significantly impact genomic stability. A relevant biological context in which G4-DNA imbalance is observed is aging and age-associated diseases [23–25]. We previously demonstrated that G4-DNA landscapes shift during cellular aging and that aged mouse brains contain more stable G4 structures than those of young mice [26]. Small molecules known as G4 stabilizers, such as pyridostatin (PDS) and pidnarulex (CX-5461), can exacerbate aging phenotypes in cultured cells, *Caenorhabditis elegans*, and mouse models [26–31].

Genomic instability, epigenetic alterations, mitochondrial impairment, disrupted protein homeostasis, cellular senescence, and metabolic dysregulation are all hallmarks of aging [32–34]. Many of these biological changes show clear associations with sex [35]. Sex-based differences are well documented in various conditions, including Alzheimer’s disease, other forms of dementia, Parkinson’s disease, stroke, and diseases affecting the heart, lungs, kidneys, and various cancers [35]. Sex hormones clearly play a significant role in regulating these differences, but they do not fully explain the extent of sexual dimorphism seen in many diseases, particularly in older individuals, where levels of gonadal hormones decline in both sexes [36]. Beyond hormonal influences, an individual’s chromosomal sex is gaining recognition as a critical determinant in aging biology and in the development of age-related neurodegenerative disorders [35, 37, 38]. For example, genes located on the X chromosome may influence susceptibility or resilience to age-associated neurodegenerative diseases in males, females, or both [39–42]. Despite these insights, the molecular roles of X- and Y-chromosome-linked mechanisms in regulating neuronal function during aging remain largely unclear.

In this study, we investigated neuronal responses to the small-molecule G4 ligand quarfloxin (CX-3543), which accumulates in the nucleolus and stabilizes G4-DNA, particularly within ribosomal DNA, thereby inducing nucleolar stress [43, 44]. We show that neuronal nucleoli are enriched in G4 structures and that quarfloxin treatment induces DNA double-strand breaks (DSBs) throughout the nucleus, with prominent enrichment in the nucleolus. Proteomic analysis of quarfloxin-treated mouse cortical neurons revealed significant alterations in 415 proteins, affecting pathways linked to neurodegenerative diseases, including Alzheimer’s, Parkinson’s and Huntington’s diseases, and amyotrophic lateral sclerosis. Among the affected proteins, we identified a nucleic acid-binding network that includes established G4-interacting proteins such as positive coactivator of transcription 4 (PC4), fused in sarcoma (FUS), heterogeneous nuclear ribonucleoprotein A1 (hnRNPA1), eukaryotic translation elongation factor 1 alpha 1 (Eef1A1), and ribosomal protein S20 (RPS20), which are all known to bind to G4s [45–48], as well as the DEAD-box helicase DDX3X.

DDX3X is an X-linked, ubiquitously expressed helicase with established roles in RNA metabolism, translational control, and cellular stress responses, and has been shown to resolve G4-RNA structures [49–55]. To examine the functional consequences of elevated DDX3X, we overexpressed DDX3X and its Y-linked homolog DDX3Y in cultured female and male neurons and performed RNA sequencing to define transcriptional responses. Both helicases modulated gene networks associated with DNA damage signaling, inflammation, cell cycle regulation, and stress-associated pathways, with notable sex-dependent effects. RNA-seq data further indicated sex-dependent splicing changes associated with DDX3X and DDX3Y expression. We also found that DDX3X expression and nuclear foci formation were increased in neurons from aged female human brains, with further elevation in female brains with Alzheimer’s disease. Our data identify DDX3X and DDX3Y as components of the neuronal response to genotoxic nucleolar stress. The induction of DDX3X and DDX3Y is accompanied by increased expression of DNA damage response genes and activation of inflammatory and cell-cycle-related mechanisms. In post-mitotic neurons, persistent activation of these pathways is often associated with dysfunction. Our findings identify previously unrecognized G4-dependent mechanisms that shape neuronal stress responses and contribute to the molecular regulation of neuronal function during brain aging, neurodegeneration, and sex-specific vulnerability.

## Results

### Stabilization of G-quadruplex structures induces nucleolar DNA damage in neurons

**Fig. 1:**
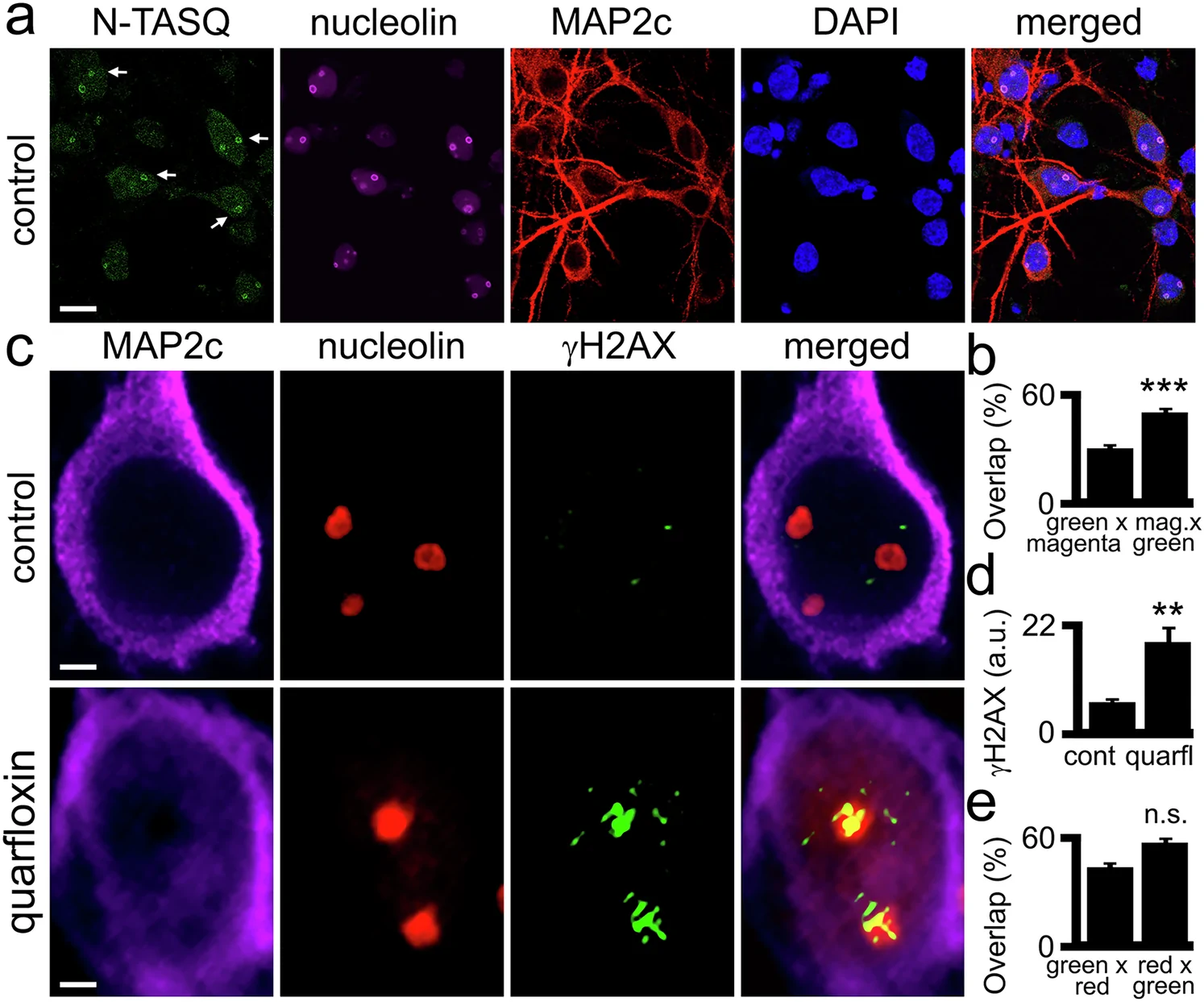
Quarfloxin (CX3543) promotes DNA DSBs in the nucleus with enrichment in the nucleolus. **a** Primary rat cortical neurons were fixed and stained with 20 μM N-TASQ, antibodies against nucleolin and MAP2c, and the nuclear dye DAPI, followed by confocal microscopy. N-TASQ-positive foci are visible in the nucleus (arrows). Scale bar, 2 μm. **b** Colocalization of N-TASQ and nucleolin signals was measured using the EzColocalization plugin in ImageJ/Fiji. Eighty-six neurons were analyzed from four independent experiments. *p* < 0.0001 (t-test). **c** Primary cortical neurons were treated with vehicle or 2 μM quarfloxin overnight, fixed, stained with antibodies against MAP2c, nucleolin, and γH2AX, and imaged by confocal microscopy. γH2AX-positive *foci* frequently colocalize with nucleolin. Scale bar, 2 μm. **d** Quantification of γH2AX levels in control neurons and neurons treated with 2 μM quarfloxin overnight. *p* = 0.002 (t-test). **e** Colocalization of nucleolin and γH2AX signals was measured using the EzColocalization plugin in ImageJ/Fiji. Ns non-significant. Eighty-six neurons were analyzed from four independent experiments in (**d**, **e**).

G4 structures are enriched in G-rich genomic regions, including ribosomal DNA within the nucleolus [56]. To determine whether neuronal nucleoli contain G4 structures, we stained primary mouse cortical neurons with the fluorescent G4 probe N-TASQ, a twice-as-smart G4 probe that functions both as a smart G4 ligand and a smart fluorescent reporter [57], together with the immunodetection of nucleolin, a nucleolar marker [58]. N-TASQ signal partially colocalized with nucleolin, indicating that neuronal nucleoli are enriched in G4 structures (Fig. 1a, b). We next examined whether pharmacological stabilization of G4 structures induces DNA damage in neurons. Cultured cortical neurons were treated with quarfloxin, and this treatment produced widespread γH2AX-positive DNA double-strand breaks throughout the nucleus, with prominent enrichment within nucleoli (Fig. 1c–e). These observations indicate that stabilization of G4 structures induces nucleolar DNA damage in neurons.

### Proteomic profiling identifies DDX3X as a stress-responsive helicase

**Fig. 2:**
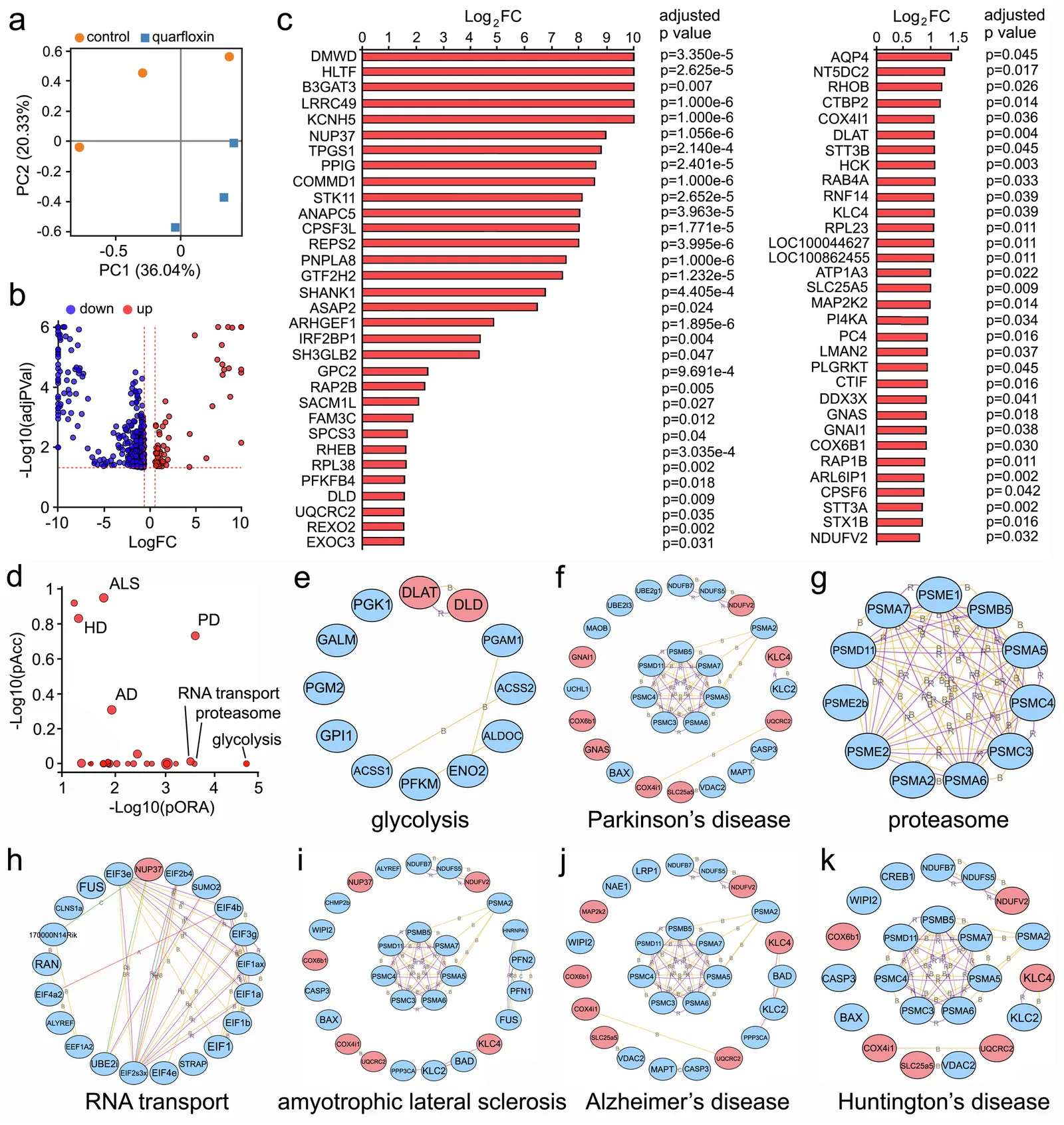
Quarfloxin alters the neuronal proteome. **a** Principal component analysis (PCA) of proteomic profiles from three vehicle-treated and three quarfloxin-treated mouse neuronal samples (2 μM overnight). Each dot represents one sample. The percentages indicate the variance explained by each principal component. Orange indicates control samples. Blue indicates quarfloxin-treated samples. **b** Volcano plot showing 415 proteins altered by quarfloxin treatment. Statistical significance is represented as the negative log10 of the *p-*value. More significant proteins appear higher on the y-axis. The dotted lines indicate thresholds for identifying altered proteins: 0.6 for expression change and 0.05 for significance. Upregulated proteins are shown in red. Downregulated proteins are shown in blue. **c** Sixty-four proteins were upregulated by quarfloxin treatment. The thresholds used were 0.6 for expression change and 0.05 for significance. **d** Pathway perturbation versus over-representation analysis showing pathways affected by 2 μM quarfloxin treatment overnight in primary neurons. Over-representation is plotted on the x-axis (pORA). Pathway perturbation is plotted on the y-axis (pAcc). Each dot represents one pathway. Dot size reflects the number of genes in that pathway. **e** The glycolysis pathway (KEGG:00010) is affected by quarfloxin. **f** The Parkinson’s disease pathway (KEGG:05012) is affected by quarfloxin. **g** The proteasome pathway (KEGG:03050) is affected by quarfloxin. **h** The RNA transport pathway (KEGG:03013) is affected by quarfloxin. **i** The amyotrophic lateral sclerosis pathway (KEGG:05014) is affected by quarfloxin. (**j**) The Alzheimer’s disease pathway (KEGG:05010) is affected by quarfloxin. **k** Huntington’s disease pathway (KEGG:05016) is affected by quarfloxin.

To gain insight into physiologically relevant pathways affected by G4 stabilization, we performed proteome-wide analysis of primary mouse cortical neurons treated with quarfloxin (mixed male and female cultures). Quarfloxin significantly altered the abundance of 415 out of 5,203 detected proteins (*ca*. 8%, log_2_FC ≥ 0.585, adjusted *p* < 0.05), corresponding to a ≥ 1.5-fold change (Fig. 2a, c and Supplementary Data 1). Among these, 64 proteins (*ca*. 15%) were upregulated (Fig. 2b, c) and 351 proteins (*ca*. 85%) were downregulated (Supplementary Fig. 1), indicating notable changes in the neuronal proteome in response to G4 stabilization by quarfloxin.

To understand the biological implications of these changes, we analyzed the proteomics data using probabilistic over-representation analysis (pORA) with KEGG pathways and Gene Ontology (GO) annotations using iPathwayGuide [59–61]. This analysis identified 22 KEGG pathways significantly affected, notably associated with neurodegenerative diseases, which were enriched, including Alzheimer’s, Parkinson’s, Huntington’s diseases, and amyotrophic lateral sclerosis. Additional affected pathways included glycolysis/gluconeogenesis, proteasome function, RNA transport, base excision repair, glutathione metabolism, pyruvate metabolism, PPAR signaling, antigen processing and presentation, and prion disease (Fig. 2d–k). In parallel, GO analysis identified 464 enriched biological processes among altered proteins. These included neuron-specific processes such as neuronal differentiation, memory, and central nervous system development. More general biological processes were also represented, including transcription initiation by RNA polymerase II, translation, and RNA localization (Supplementary Fig. 2).

### Quarfloxin alters a network of nucleic acid-binding proteins

**Fig. 3:**
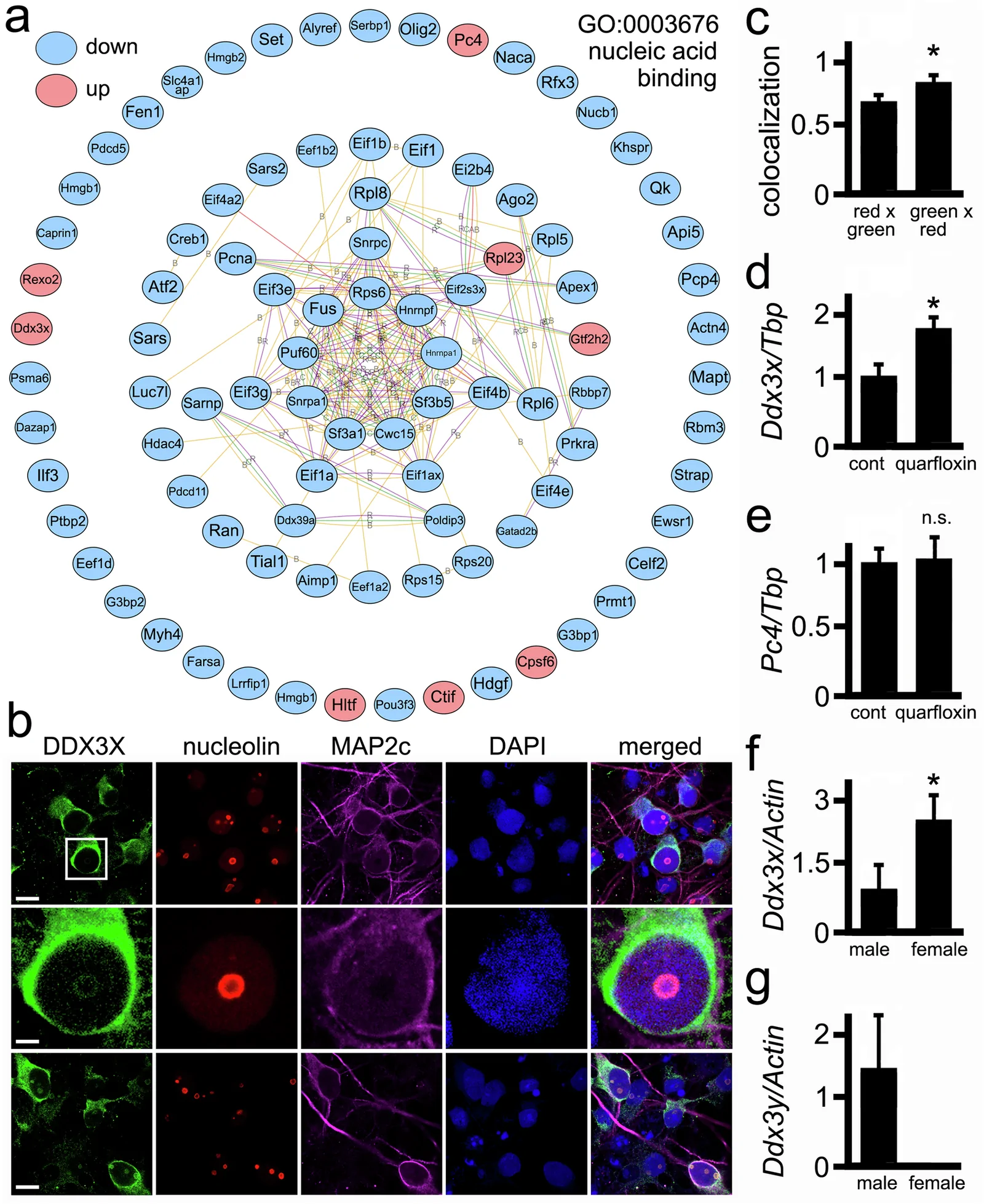
Quarfloxin alters a network of nucleic acid-binding proteins. **a** DNA damage response proteins and transcription factors are among the nucleic acid-binding proteins affected by quarfloxin (GO:0003676). These proteins include positive coactivator of transcription 4 (PC4), fused in sarcoma (FUS), heterogeneous nuclear ribonucleoprotein A1 (hnRNPA1), eukaryotic translation elongation factor 1 alpha 1 (Eef1A1), and ribosomal protein S20 (RPS20), which are known G-quadruplex-binding proteins. DDX3X, a ubiquitous helicase, was also upregulated following quarfloxin treatment. **b** Primary cortical neurons were stained with antibodies against DDX3X (green), nucleolin (red), MAP2c (magenta), and DAPI. Images were acquired by confocal microscopy. Scale bar, 5 μm. The enlarged image shows a nuclear DDX3X signal with nucleolar enrichment that colocalizes with nucleolin. Scale bar, 5 μm. Neurons in the lower panel show higher levels of nucleolar DDX3X. DDX3X-positive structures are visible in neuronal nucleoli and colocalize with nucleolin. Scale bar, 5 μm. **c** Colocalization of DDX3X and nucleolin signals. *p* = 0.037 (t-test). Eighty neurons were analyzed from four independent experiments. **d** Primary cortical neurons were treated with vehicle or 2 μM quarfloxin overnight. Ddx3x expression was measured by quantitative RT-PCR relative to Tbp. *p* = 0.02 (t-test). Data were pooled from four independent experiments. **e** Primary cortical neurons were treated with vehicle or 2 μM quarfloxin overnight. *Pc4* expression was measured by quantitative RT-PCR relative to *Tbp*. Ns not significant. Data were pooled from four independent experiments. **f** Expression of *Ddx3x* measured by quantitative RT-PCR relative to *Actin* in male and female primary cortical neurons. *p* = 0.0471 (t-test). Data were pooled from eight independent experiments. **g** Expression of *Ddx3y* measured by quantitative RT-PCR relative to *Actin* in male and female neurons. Data were pooled from eight independent experiments.

Nucleic acid-binding proteins were among the most significantly affected by quarfloxin treatment (GO:0003676). This group included several factors involved in DNA damage repair, such as FUS, APEX1, FEN1, PCNA, HMGB1, and HMGB2, as well as transcriptional regulators including PC4, HDAC4, KAT8, and ATF2 (Fig. 3a). The network also contained proteins previously reported to interact with G4s, including PC4, FUS, hnRNPA1, Eef1A1 and RPS20 (Fig. 3a). Earlier, we demonstrated that PC4 binds to a G4 identified in the *Atg7* autophagy gene [26]. It is not clear, however, if these proteins bind to nucleolar G4s, but fluorescently tagged PC4 does form intranuclear puncta and colocalizes with nucleolin, indicating that the protein is enriched in the nucleolus (Supplementary Fig. 3a–e).

Quarfloxin treatment also increased the abundance of DDX3X (Fig. 3a). Immunostaining revealed strong cytoplasmic localization of DDX3X in neurons together with distinct nucleolar puncta that colocalized with nucleolin (Fig. 3b, c). To investigate a mechanism of this upregulation, we treated cultured primary cortical neurons with quarfloxin, extracted mRNA, and performed qPCR analysis for *Ddx3x* and *Pc4* (as a control). *Ddx3x* transcripts increased following treatment, whereas *Pc4* mRNA levels remained unchanged (Fig. 3d, e). These findings suggest that neurons respond to G4 stabilization by inducing DDX3X expression, potentially as part of a compensatory response to DNA damage. Based on these findings, DDX3X was prioritized because it is a known G4-resolving helicase and was upregulated following quarfloxin treatment.

### DDX3X and DDX3Y regulate transcription and splicing in neurons with sex differences

We next examined the Y-linked homolog of DDX3X, DDX3Y. Although DDX3Y is best known for its role in spermatogenesis, it has also been detected at low levels in the central nervous system [62]. To determine whether *Ddx3y* is expressed in our neuronal model, we analyzed cortical neurons derived from individual male and female mouse embryos that were genotyped for the Y-linked *Sry* gene. As expected, *Ddx3x* transcripts were detected in neurons from both sexes, with higher expression in female cultures, whereas *Ddx3y* expression was restricted to male neurons (Fig. 3f, g; Supplementary Fig. 4a, b). Using RNAscope in *postmortem* human brain samples, we also detected DDX3Y transcripts in male brains (Supplementary Fig. 4c–e).

**Fig. 4:**
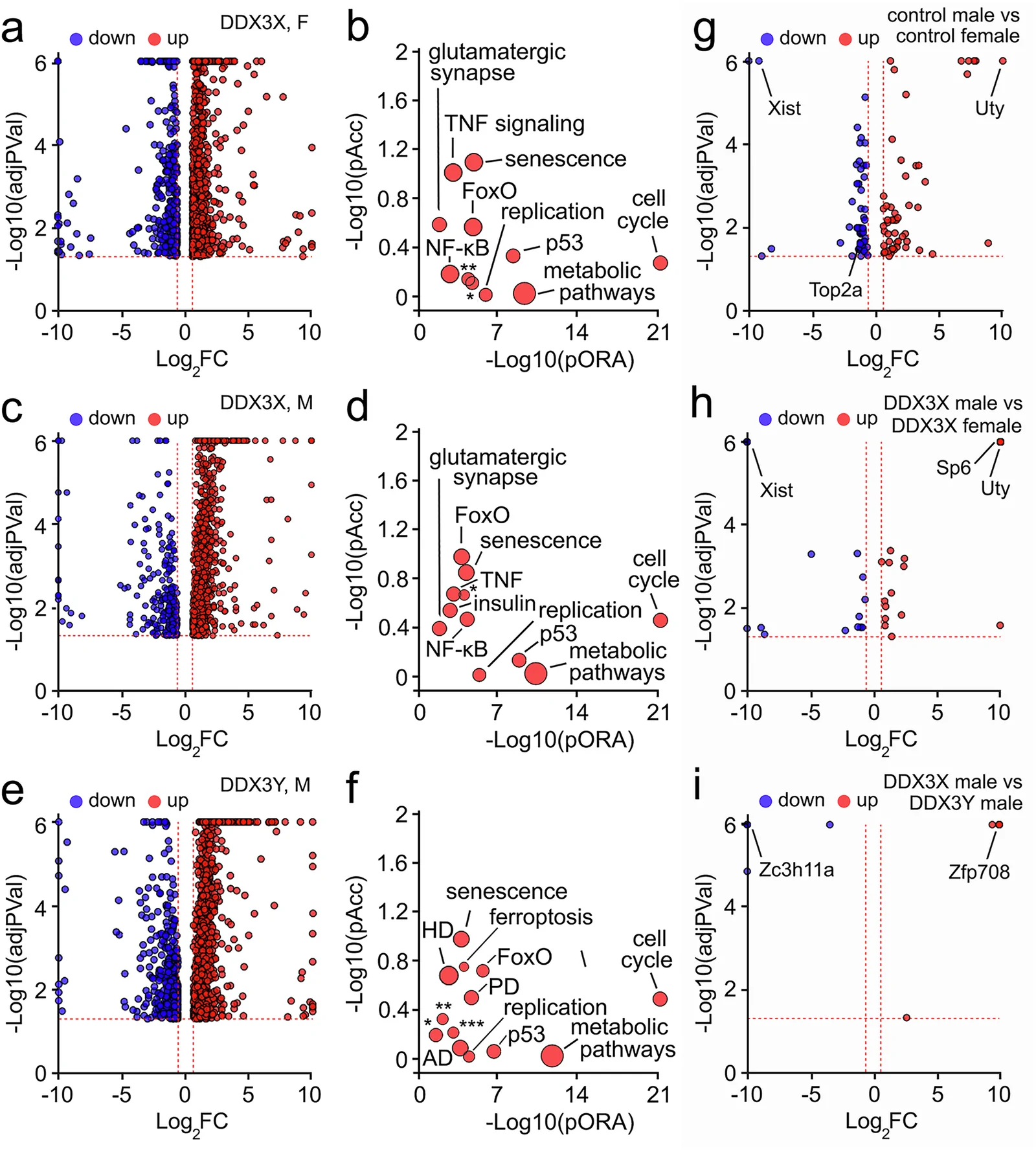
DDX3X and DDX3Y alter transcription in primary female and male cortical neurons. **a** Female neurons were infected with control mScarlet or DDX3X-mScarlet virus. mRNA was isolated and analyzed by RNA-seq. The volcano plot shows 1226 differentially expressed genes. **b** Pathway analysis showing networks affected by DDX3X in female neurons. The asterisks indicate the serotonergic synapse pathway (*) (KEGG:04726) and the GABAergic synapse pathway (**) (KEGG:04727). **c** Male neurons were infected with control mScarlet or DDX3X-mScarlet virus. mRNA was isolated and analyzed by RNA-seq. The volcano plot shows 1225 differentially expressed genes. **d** Pathway analysis showing networks affected by DDX3X in male neurons. The asterisk indicates the ferroptosis pathway (*) (KEGG:04216). **e** Male neurons were infected with control mScarlet or DDX3Y-mScarlet virus. mRNA was isolated and analyzed by RNA-seq. The volcano plot shows 1671 differentially expressed genes. **f** Pathway analysis showing networks affected by DDX3Y in male neurons. The asterisks indicate the amyotrophic lateral sclerosis pathway (***) (KEGG:05014), insulin signaling pathway (**) (KEGG:04910), and NF-κB signaling (*) (KEGG:04064). In panels (a), (c), and (e), five neuronal samples per condition were used. Statistical significance is represented by the negative log10 of the p-value. More significant genes appear higher on the y-axis. Dotted lines indicate thresholds of 0.6 for expression change and 0.05 for statistical significance. Upregulated genes are shown in red. Downregulated genes are shown in blue. In (**b, d, f**), each dot represents one pathway. Dot size reflects the number of genes in the pathway. **g** Male and female neurons show sex differences in transcriptomes. Volcano plot showing 108 differentially expressed genes in male and female cultured neurons. **h** Volcano plot showing 39 differentially expressed genes in male and female neurons expressing DDX3X. **i** Volcano plot showing 11 differentially expressed genes in male neurons expressing either DDX3X or DDX3Y.

To examine the functional consequences of increased helicase expression, we overexpressed DDX3X in male and female cortical neurons and DDX3Y in male neurons only, followed by RNA sequencing (Fig. 4). qPCR confirmed comparable expression of the viral transgenes in cultures infected at the same multiplicity of infection, supporting the interpretation of sex-dependent transcriptional effects (Supplementary Fig. 5).

DDX3X overexpression altered the expression of over 1,200 genes in both female and male neurons. The affected genes were enriched in pathways associated with cellular stress responses, including DNA damage signaling, cell cycle regulation, senescence, FoxO signaling, inflammatory pathways, and neuronal signaling networks (Fig. 4a–d; Supplementary Figs. 6, 7). Many of these pathways were shared between male and female neurons, although the magnitude and composition of the transcriptional response differed between sexes.

Overexpression of DDX3Y in male neurons produced a similarly broad transcriptional response, affecting more than 1600 genes. These changes overlapped with many pathways regulated by DDX3X, including stress responses, senescence, FoxO signaling, inflammatory signaling, DNA damage response, and neuronal functional networks. However, several pathways appeared more prominently associated with DDX3Y, including ferroptosis and pathways linked to ferroptosis and neurodegenerative diseases such as Alzheimer’s and Parkinson’s diseases, as well as amyotrophic lateral sclerosis (Fig. 4e, f**;** Supplementary Figs. 6, 7).

Baseline transcriptomes of male and female neurons also showed intrinsic sex differences, with 108 genes differentially expressed between the two conditions (Fig. 4g–i). Baseline sex differences were accounted for by comparing matched cultures and normalized expression levels. Overexpression of DDX3X or DDX3Y further amplified these transcriptional differences, suggesting that DDX3 helicases contribute to sex-specific regulatory mechanisms in neurons.

Finally, we examined whether DDX3 helicases influence RNA processing. Analysis of alternative splicing events revealed that DDX3X and DDX3Y significantly altered multiple splicing classes, including alternative splice sites, mutually exclusive exons, retained introns, and exon skipping. These changes showed clear sex-dependent patterns and are consistent with previously reported roles of DDX3 helicases in RNA metabolism (Supplementary Fig. 8; Supplementary Data 2).

### DDX3X and DDX3Y modulate DNA damage response gene networks

**Fig. 5:**
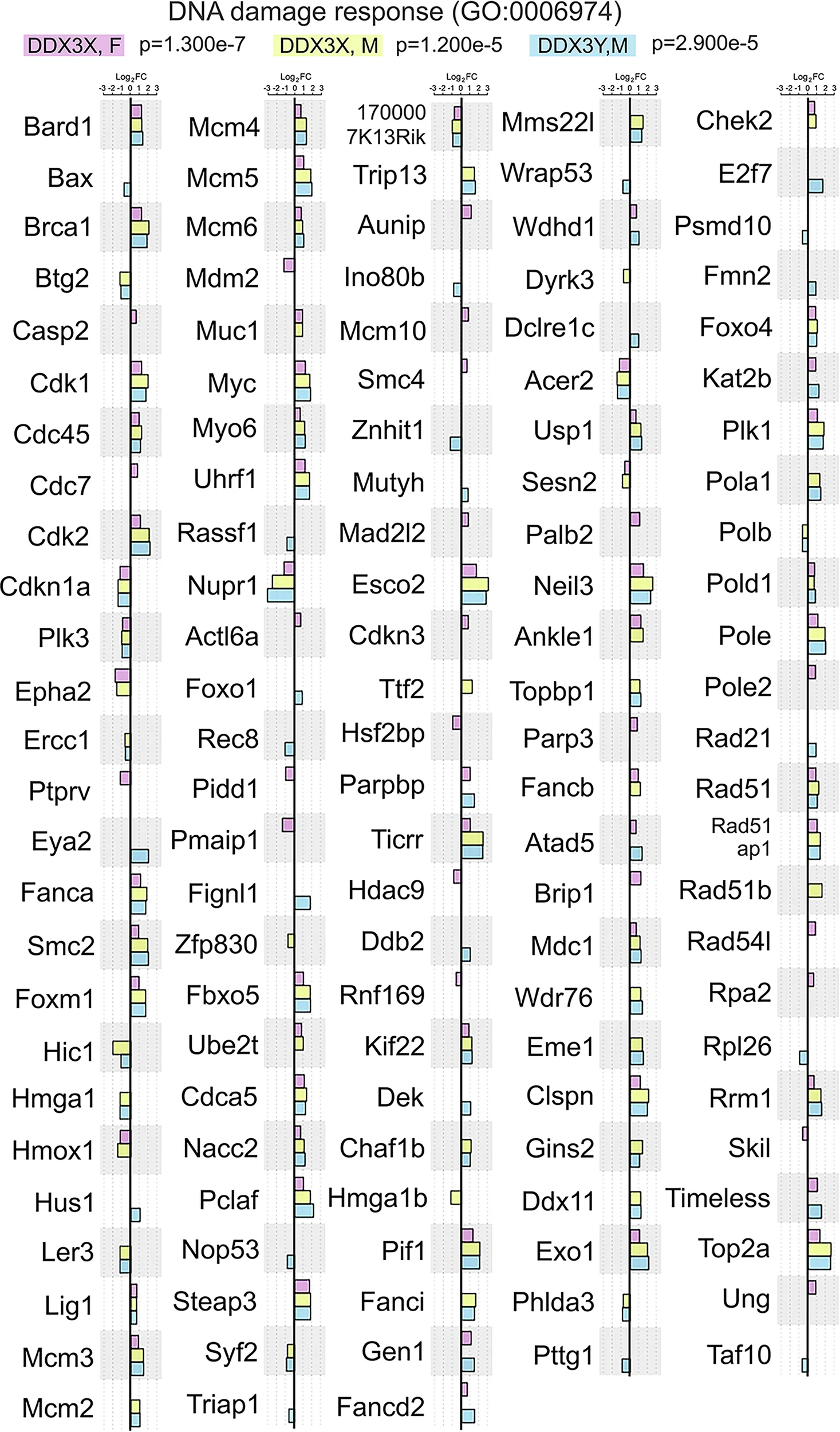
DDX3X and DDX3Y regulate the DNA damage response gene network in male (DDX3Y only) and female neurons. DDX3X alters DNA damage response genes in female neurons (pink), DDX3X in male neurons (yellow), or DDX3Y in male neurons (blue) (GO:0006974).

Our proteomic data indicated that DDX3X is part of a network of nucleic acid-binding proteins altered by quarfloxin treatment, a condition that induces DNA damage in neurons (Fig. 3a). RNA-seq analysis further showed that overexpression of either DDX3X or DDX3Y affects transcriptional networks involved in DNA damage responses (Fig. 5). Several genes associated with genome maintenance and G4-related DNA processing were upregulated, including *Pif1* (G4-DNA helicase), *Neil3* (DNA glycosylase), *Top2a* (DNA topoisomerase), *Pole* (catalytic subunit of DNA polymerase epsilon), *Brca1* (breast cancer type 1 susceptibility protein), *Fanca* (Fanconi anemia complementation group A), *Smc2* (structural maintenance of chromosomes protein 2), *Rad51* (DNA repair protein RAD51 homolog 1), and *Exo1* (5′ to 3′ exonuclease and RNase). Several components of the minichromosome maintenance (Mcm) complex, including *Mcm3*, *Mcm4*, *Mcm5*, and *Mcm10*, were also upregulated. In male neurons, DDX3Y, but not DDX3X, induced *Fignl1* (fidgetin-like 1), a gene implicated in meiotic double-strand break repair and spermatogenesis [63] (Fig. 5; Supplementary Data 3). These results indicate that DDX3X and DDX3Y regulate transcriptional networks associated with DNA damage response, with sex-dependent differences. Finally, we validated key RNA-seq findings by quantitative PCR, supporting the RNA-seq findings and confirming increased expression of DNA damage-associated genes (Supplementary Fig. 9).

### DDX3X and DDX3Y regulate cell cycle, inflammatory, and senescence-associated pathways

**Fig. 6:**
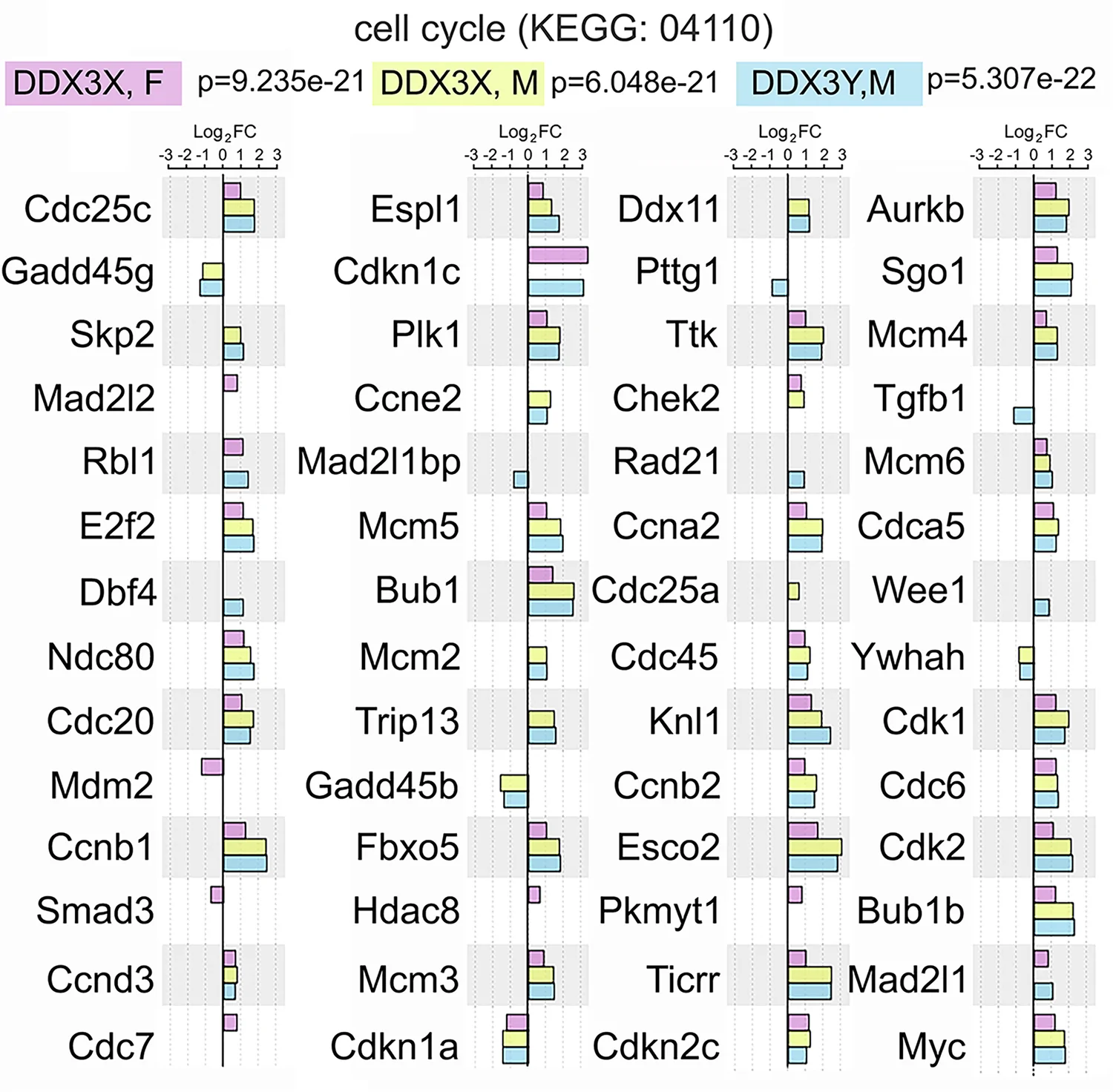
DDX3X and DDX3Y regulate the cell cycle gene network in male (DDX3Y only) and female neurons. DDX3X alters cell cycle genes in female neurons (pink), DDX3X in male neurons (yellow), or DDX3Y in male neurons (blue) (KEGG: 04110).

Previous studies have shown that DDX3X overexpression can promote inflammatory signaling in vascular cells [64]. Consistent with this, our data indicate that both DDX3X and DDX3Y modulate gene networks linked to cell cycle regulation, inflammation, and cellular senescence, suggesting that sustained elevation of these helicases may promote transcriptional pathways associated with inflammation and cellular senescence in neurons (Fig. 6; Supplementary Figs. 11, 12).

DDX3X and DDX3Y modulated the expression of genes involved in cell cycle regulation. Both helicases increased the expression of *Myc*, *Aurkb*, *Plk1*, and *Cdk1* in male and female neurons, while *Mad2l1* was selectively induced by DDX3X in female neurons. In addition, *Chek2* (checkpoint kinase 2) was upregulated by DDX3X in both sexes (Fig. 6). To assess whether DDX3X or DDX3Y expression is associated with functional changes, we examined neuronal morphology over time. Neurons expressing DDX3X or DDX3Y showed a reduction in neuronal area compared to controls, indicating a decrease in neuronal arborization associated with DDX3X and DDX3Y expression (Supplementary Fig. 10).

Within inflammatory pathways, overexpression of DDX3X or DDX3Y increased the expression of key immune-related genes, including *C3*, *Il1r1*, and *Tlr3*, in both male and female neurons. Some responses were sex-specific. *Cxcl5* was upregulated by DDX3X in female neurons, whereas *Cxcl2* was downregulated by DDX3X or DDX3Y specifically in male neurons. *Orm2* was selectively upregulated by DDX3Y, while *Cxcl3* was reduced by DDX3Y in male neurons and by both helicases in female neurons (Supplementary Fig. 11). These findings indicate that DDX3 helicases modulate inflammatory gene networks with sex-dependent effects.

Post-mitotic neurons can enter a senescence-like state, often referred to as functional or post-mitotic senescence, in which aberrant activation of cell cycle genes leads to neuronal dysfunction rather than proliferation [65–71]. Consistent with this, DDX3X and DDX3Y altered transcriptional networks associated with cellular senescence. However, these changes occurred together with reduced expression of *Cdkn1a*, a canonical senescence marker. Instead, several cell cycle regulators, including *Ccna2* (cyclin A2), *Ccnb1* (cyclin B1), *Ccnb2* (cyclin B2), and *Mybl2*, were upregulated. These findings are more consistent with activation of an abortive cell cycle re-entry program than with classical p21-mediated senescence (Supplementary Fig. 12).

DDX3X and DDX3Y also affected neuronal functional gene networks. Both helicases reduced the expression of *Npas4* (neuronal PAS domain protein 4) and *Arc* (activity-regulated cytoskeleton-associated protein), genes that play central roles in activity-dependent transcription and synaptic plasticity (Supplementary Figs. 13, 14) [72, 73]. These data suggest that persistent activation of DDX3 helicases may modulate neuronal function by altering gene networks involved in synaptic signaling, memory and behavior.

### DDX3X levels are higher in neurons of aged and Alzheimer’s disease female brains

DDX3X displays sex-dependent expression patterns. Previous studies have shown that *Ddx3x* escapes X-chromosome inactivation in female fibroblasts and immune cells and may do so during aging [74, 75]. Consistent with these observations, we found that *Ddx3x* transcripts were expressed at higher levels in cultured female neurons than in male neurons (Fig. 3f).

We next compared DDX3X protein expression in cultured male and female mouse neurons and in mouse and human brain tissues. First, we evaluated the specificity of our anti-DDX3X antibodies and determined whether they cross-react with DDX3Y. We used cultured male brain endothelial cells infected with either control mScarlet or DDX3Y-mScarlet lentivirus. In control cells, mScarlet and anti-DDX3X signals showed no correlation. In contrast, cells expressing DDX3Y-mScarlet showed a strong correlation between mScarlet and anti-DDX3X signal intensity, indicating that the DDX3X antibodies also recognize DDX3Y (Supplementary Fig. 15a, b). Second, we tested the specificity of the DDX3Y antibody using female mouse hippocampal tissue, which lacks Y-chromosome expression. These samples still showed staining with the DDX3Y antibody, likely reflecting cross-reactivity with DDX3X and preventing reliable detection of DDX3Y in male tissues (Supplementary Fig. 15c). Based on these findings, subsequent analyses focused on DDX3X in female samples lacking the Y chromosome and therefore not expressing DDX3Y.

**Fig. 7:**
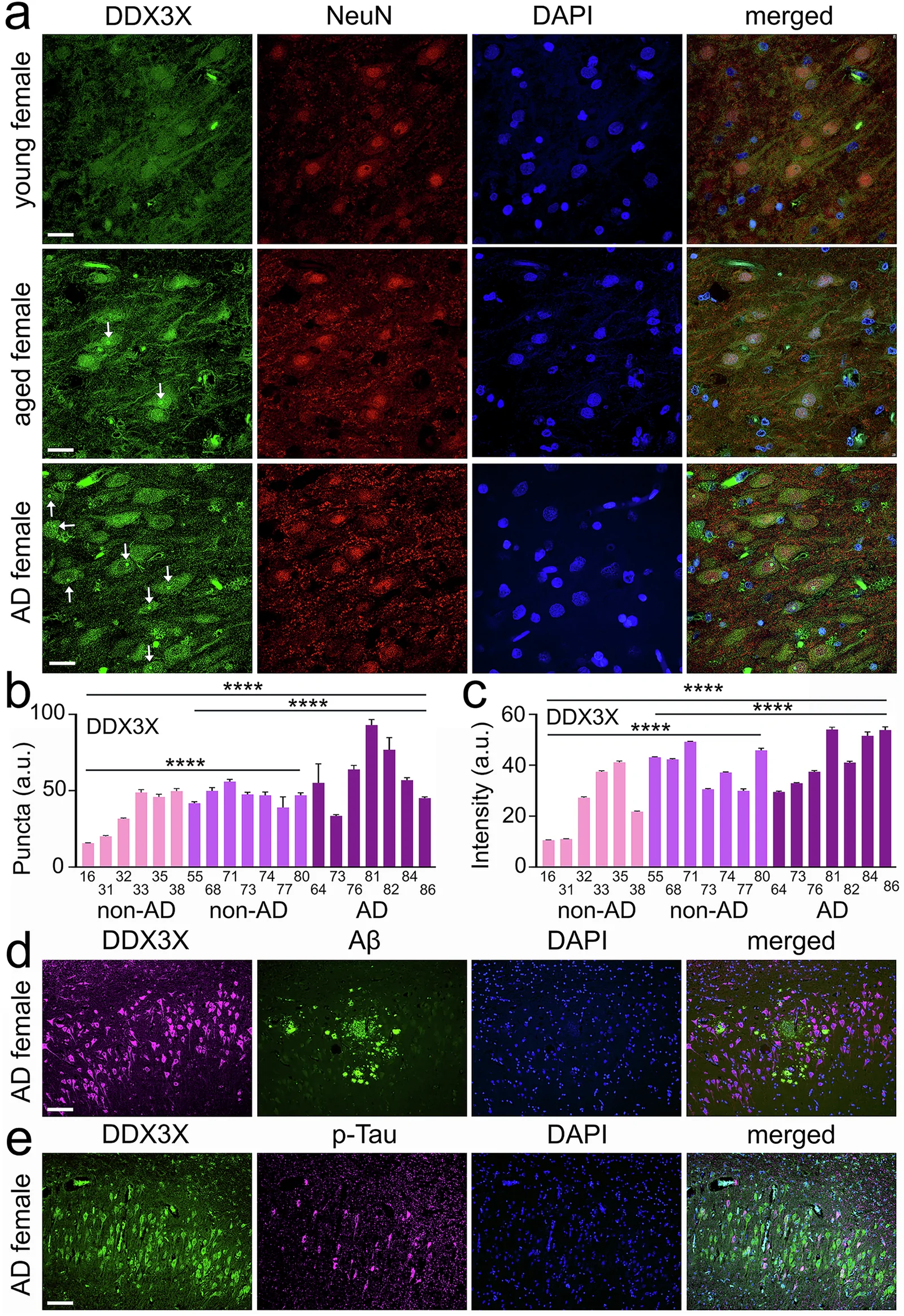
DDX3X expression in the female human hippocampus. **a** CA1 hippocampal sections from a young female (35 years), an aged female (80 years), and a female with Alzheimer’s disease (86 years) were stained with antibodies against DDX3X and NeuN and with the nuclear dye DAPI. Samples were obtained from autopsy tissue. Nuclear DDX3X-positive puncta *foci* are visible in aged and Alzheimer’s disease samples. Scale bar, 10 μm. **b** Quantification of the puncta index of DDX3X-positive foci in six young females (16, 31, 32, 33, 35, 38 years), seven aged females (55, 68, 71, 73, 74, 77, 80 years), and seven females with Alzheimer’s disease (64, 73, 76, 81, 82, 84, 86 years). See Supplementary Table 1 for neuropathological details. Approximately 400 neurons were analyzed per individual. Data were pooled for group comparison. *p* < 0.0001 (t-test). **c** Fluorescence intensity of DDX3X was measured in the same cohort of young, aged, and Alzheimer’s disease females. Approximately 400 neurons were analyzed per individual. Data were pooled for group comparison. *p* < 0.0001 (t-test). **d** CA1 hippocampal sections from a female Alzheimer’s disease case (73 years old) were stained with antibodies against DDX3X and amyloid-β (Aβ), along with the nuclear dye DAPI. Representative images show DDX3X signal and Aβ-positive plaques within the same region. Scale bar, 100 μm. **e** CA1 hippocampal sections from a female Alzheimer’s disease case (86 years old) were stained with antibodies against DDX3X and phosphorylated tau (p-Tau), along with the nuclear dye DAPI. Representative images show DDX3X signal and p-Tau-positive pathology within the same region. Scale bar, 100 μm.

**Fig. 8:**
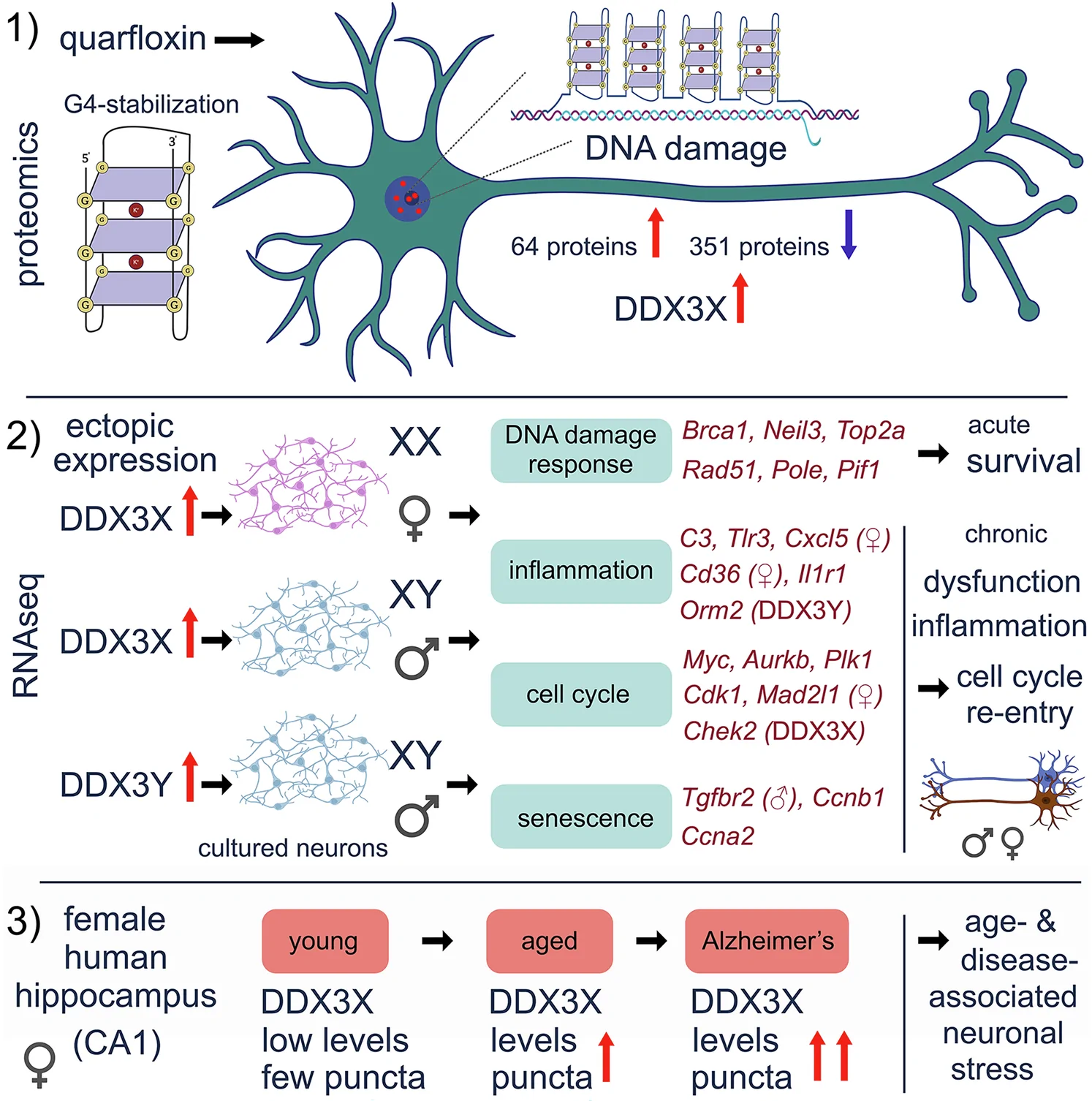
Model of DDX3X and DDX3Y function in neurons. G4 stabilization by quarfloxin induces DNA damage in neurons, particularly in the nucleolus, and alters proteomic networks linked to neurodegenerative diseases. This response includes upregulation of the G4 helicase DDX3X. DDX3X and its Y-linked homologue DDX3Y regulate gene networks involved in DNA damage response, inflammation, cell cycle, and senescence. In post-mitotic neurons, sustained activation of these pathways is associated with neuronal dysfunction. In human brains, DDX3X levels and nuclear puncta increase in neurons from aged females and are further elevated in Alzheimer’s disease.

We next examined the DDX3X protein expression in the human hippocampus from female individuals in different age groups. DDX3X frequently formed punctate structures in CA1 neurons of aged females, and this pattern was further increased in Alzheimer’s disease cases (Fig. 7a, b**;** Supplementary Table 1). Quantification showed that DDX3X levels were higher in aged female brains compared with younger individuals and were further elevated in Alzheimer’s disease (Fig. 7c). Neuropathological examination of the Alzheimer’s samples confirmed the presence of amyloid plaques (Aβ) and tau pathology, together with GFAP-positive astrocytes and IBA1-positive microglia, consistent with neuroinflammation (Fig. 7d, e**;** Supplementary Fig. 16). Correlation analyses showed that DDX3X puncta and levels were more consistently associated with markers of gliosis (GFAP and IBA1) than with Aβ or phosphorylated tau, suggesting a closer relationship with neuroinflammatory features of Alzheimer’s disease (Supplementary Fig. 16). Because DDX3X is induced by G4 stabilization and DNA damage (Fig. 3a, d), both of which are prominent features of Alzheimer’s disease pathology [76–78]. These findings suggest a role for DDX3X in neuronal responses to genomic stress in the aging and diseased brain (Fig. 8).

## Discussion

In this study, we show that pharmacological stabilization of G4 structures with quarfloxin induces nuclear DNA damage in neurons, with DSBs, particularly enriched in the nucleolus. Proteomic profiling identified a network of proteins altered by quarfloxin treatment (*ca*. 8% of the proteome), including 64 upregulated and 351 downregulated proteins. Many of these protein networks overlap with pathways previously reported in neurodegenerative diseases such as Alzheimer’s, Parkinson’s, Huntington’s diseases, and amyotrophic lateral sclerosis. We also identified a nucleic acid-binding protein network containing PC4, FUS, hnRNPA1, Eef1A, and RPS20, all of which are known G4-binding proteins [45–48]. supporting the biological relevance of our dataset.

Among the proteins upregulated by quarfloxin, we identified the G4 helicase DDX3X, encoded on the X chromosome. To examine the functional consequences of DDX3X upregulation, we overexpressed DDX3X and its Y-linked homolog DDX3Y in cultured female and male mouse neurons and performed RNA-seq analysis. Both helicases altered gene networks associated with DNA damage response, inflammation, cell cycle-related programs, and senescence-associated pathways, with clear sex-dependent differences. In addition, DDX3X and DDX3Y influenced alternative splicing in a sex-specific manner, further supporting their roles in gene regulation. We also observed increased expression and nuclear accumulation of DDX3X in neurons from older female human brains compared with younger individuals, with further elevation in females with Alzheimer’s disease. Taken together, these observations indicate an association between DDX3X and DDX3Y regulation and sex-specific neuronal stress responses observed during aging and neurodegenerative disease.

DDX3 belongs to the DEAD-box helicase family and is encoded by two genes in mammals: *Ddx3x* on the X chromosome and *Ddx3y* on the Y chromosome. Although DDX3X and DDX3Y share 92% homology [79, 80]. They differ in expression patterns and regulatory roles. DDX3Y was initially described as restricted to spermatocytes, where it is required for spermatogenesis [81]. whereas DDX3X is ubiquitously expressed and regulates transcription, RNA splicing, and translation [55, 82]. DDX3X has been extensively studied in cancer biology [82], and mutations in DDX3X have also been linked to a rare neurodevelopmental disorder, underscoring the sensitivity of neuronal systems to altered DDX3X regulation [55, 83]. Notably, *Ddx3x* escapes X-chromosome inactivation in certain cell types and has been reported to do so during aging in mice, suggesting a potential basis for sex-dependent effects [74, 75].

Our data support these observations by showing that female neurons express higher levels of *Ddx3x* than male neurons in culture and that DDX3X forms nuclear puncta in neurons from human Alzheimer’s disease brains, with more pronounced accumulation in females. Although the functional significance of these nuclear structures remains to be defined, they may reflect sites of transcriptional and splicing regulation, DNA damage response, or both. Given the major roles of genomic maintenance and transcriptional control in neuronal vulnerability, these findings support a potential mechanism linking DDX3X regulation to sex-specific features observed in Alzheimer’s disease and related disorders.

DDX3Y, long thought to be restricted to the male germline, has been detected at low levels in the central nervous system [62]. In male cortices lacking *Ddx3x*, *Ddx3y* expression is upregulated, suggesting a transcriptional adaptation and potential neuronal expression [84]. Although DDX3X and DDX3Y can be functionally redundant in some contexts, a 60% difference in their N-termini confers distinct roles in translational regulation and stress granule dynamics [85]. Notably, DDX3Y has been reported to promote the aggregation of FUS and TDP-43 more strongly than DDX3X in non-neuronal cells, suggesting that these helicases may differentially influence protein homeostasis [85]. Whether DDX3Y contributes to sex-specific aspects of neuronal aging and neurodegeneration remains an open question, particularly given age-associated Y-chromosome loss and epigenetic alterations reported in male cells [86–88], a process that may also occur in neurons [88, 89]. Our data reveal distinct transcriptional and splicing effects of DDX3X and DDX3Y in neurons, supporting non-redundant functions in the brain.

Neurons are particularly vulnerable to genomic stress due to their long lifespan and high levels of transcriptional and metabolic activity [90]. We previously showed that G4 structures accumulate in the aging mouse brain and that excessive G4 stabilization disrupts genomic stability in multiple brain cell types^2630, 92^. G4 structures can interfere with transcription and topoisomerase activity, ultimately leading to DNA damage when not properly regulated [21, 91]. In the present study, quarfloxin-induced G4 stabilization promoted DNA damage, with enrichment in the nucleolus. This pattern is consistent with stabilization of G4s within ribosomal DNA, one of the most actively transcribed and damage-prone regions of the genome.

The nucleolus serves as both a center for ribosome biogenesis and a hub for DNA damage response signaling. The nucleolus houses ribosomal DNA (rDNA), RNA polymerases I–III, pre-rRNA, noncoding RNAs (ncRNAs), promoter-associated RNAs (pRNAs), and various ribonucleoprotein complexes [92, 93]. Since rRNA accounts for approximately 80% of the total cellular RNA, rDNA represents the most actively transcribed region of the genome [92]. Given the high energy demands of rRNA synthesis, rDNA transcription is downregulated during cellular stress, linking nucleolar activity to pathways governing growth, proliferation, and longevity [94, 95]. The nucleolus is particularly vulnerable to DNA damage due to the repetitive nature of rDNA, categorized as hard-to-replicate sequences, and its intense transcriptional activity [96]. As a result, it functions both as a hotspot for genomic instability and a central hub for the DDR, with over 160 known DNA repair proteins localizing to this structure [97]. Therefore, the enrichment of DNA damage within the nucleolus following quarfloxin treatment resembles stress responses reported in aging cells and may contribute to neuronal vulnerability under conditions of sustained genomic stress.

Ectopic expression of DDX3X and DDX3Y induced transcriptional programs associated with DNA damage response, including upregulation of genes involved in DNA repair such as *Pif1* (G4-DNA helicase), *Neil3* (DNA glycosylase), *Top2a* (DNA topoisomerase), *Pole* (catalytic subunit of DNA polymerase epsilon), *Brca1* (breast cancer type 1 susceptibility protein), *Fanca* (Fanconi anemia, complementation group A), *Smc2* (structural maintenance of chromosomes protein 2), *Rad51* (DNA repair protein RAD51 homolog 1), and *Exo1* (5’ to 3’ exonuclease and RNase). These changes represent compensatory responses aimed at maintaining genomic integrity. However, elevation of DDX3X and DDX3Y was also associated with increased expression of inflammatory genes, such as *C3* and *Tlr3*, in both male and female neurons, as well as cell-cycle-associated genes, a phenomenon observed in Alzheimer’s disease brains [98]. Our data align with previous findings that link DDX3X to aberrant activation of cyclin-dependent kinases in a DDX3X syndrome mouse model [84]. Our findings suggest that while DDX3X and DDX3Y contribute to maintaining genomic integrity in neurons, their involvement in inflammation and cell cycle dysregulation may exacerbate neurodegeneration in Alzheimer’s disease, particularly when their upregulation is chronic (Fig. 8). Future studies should explore the balance between these opposing functions to determine whether modulating DDX3X and DDX3Y activity could provide therapeutic benefits for neurodegenerative diseases.

Our findings identify DDX3X and DDX3Y as components of a neuronal stress response triggered by G4-associated DNA damage. These helicases act as regulators of multiple gene networks, including those governing DNA damage response, inflammation, cell cycle progression, and senescence-associated pathways, with clear sex-dependent differences. Since chronic DNA damage stress is a well-established contributor to neurodegeneration, our findings offer valuable insight into molecular pathways that may underlie neurodegenerative disorders.

## Materials and methods

### Chemicals and plasmids

Antibodies against MAP2c (1:1000) were obtained from Novus Biologicals (#NB300-213). Antibodies against nucleolin (1:1000) were from Cell Signaling Technology (#14574). Antibodies against γH2AX (1:500) were from Millipore (JBW301, #05-636). Antibodies against NeuN (1:1000) were from Millipore (#ABN91). Antibodies against DDX3X (anti-mouse, 1:500) were from Abcam (#ab196032). Antibodies against DDX3X, anti-rabbit (1:500), were from Proteintech (#11115-1-AP). For co-staining experiments with Aβ (MOAB-2) and p-Tau, the DDX3X antibody from Proteintech was used to avoid host species overlap. Antibodies against DDX3Y (1:500) were from Thermo Scientific (#PA522050). Antibodies against Aβ (MOAB-2) (1:500) were from Novus Biologicals (#NBP2-13075AF488). Antibodies against IBA1 (1:500) were from Novus Biologicals (#NB100-1028SS). Antibodies against GFAP (1:500) were from Novus Biologicals (#NB100-53809). Antibodies against p-Tau (1:500) were from Invitrogen (#MN1020). Secondary antibodies included anti-mouse Alexa Fluor 488 (1:1000, #A11001), anti-rabbit Alexa Fluor 546 (1:1000, #A11010), anti-goat Alexa Fluor 546 (1:1000, #A32814), and anti-chicken Alexa Fluor 647 (1:1000, #A21449), all obtained from Life Technologies. N-TASQ was synthesized as described previously [57, 99]. The nucleic acid stains SYTOX Deep Red (#P36990) and DAPI (#62248) were purchased from Thermo Fisher Scientific. Quarfloxin (CX-3543) was obtained from AdooQ BioScience (#A12380-25). The G4P sequence was described previously by the Tan laboratory [100]. (see Acknowledgments). The plasmids pRP-CAG-TagBFP, pRP-CAG-PC4-TagBFP, pRP-CAG-PC4-mScarlet, and pRP-CAG-DDX3X-mScarlet were obtained from VectorBuilder. The lentiviruses pLV[Exp]-EF1A>mDdx3x, pLV[Exp]-EF1A>mScarlet and pLV[Exp]-EF1A>mDdx3y were generated with VectorBuilder. RNAscope probes included Hs-DDX3Y-C1 (#892681), Hs-DDX3X-O2-C2 (#1823271-C2), and Hs-RBFOX3-C3 (NeuN, #415591-C3), all from Advanced Cell Diagnostics. TSA-Vivid 650 (1:3000) was obtained from Bio-techne (#7527). Opal 570 dye (1:1500, #FP1488001KT) and Opal 520 dye (1:1500, #FP1487001KT) were obtained from Akoya Biosciences.

### Cell culture

Brain cortices from rat or mouse embryos (E17–18) were dissected, dissociated, and plated on 12-well tissue culture plates (4 × 10^5^ cells per well). Plates were coated with poly-D-lysine (Sigma-Aldrich, P6407) as described previously [26]. Primary cortical neurons were maintained in Neurobasal Plus Medium (Gibco, #21103049) supplemented with B-27 (Gibco, #17504001), GlutaMAX (Gibco, #35050061), and penicillin-streptomycin (Gibco, #15140122). Neurons were used at 14 days in vitro. Primary rat cultures at 4 days in vitro were transfected with Lipofectamine 2000 (Thermo Fisher Scientific, #11668019) using 1–2 μg of plasmid DNA expressing pRP-CAG-TagBFP, pRP-CAG-PC4-TagBFP, pRP-CAG-PC4-mScarlet, and pRP-CAG-DDX3X-mScarlet, per well, as described previously [26]. The rat neurons were collected and processed for proteomic analysis. To examine sex differences, cortices from individual mouse embryos (E17–18) were dissected and cultured separately. Mouse neurons were seeded on 12-well plates (4 × 10^5^ cells per well). Mouse embryo tails were collected for genotyping to determine sex using PCR detection of the testis-determining gene *Sry*. Primary mouse neuronal cultures were infected at 14 days in vitro with lentiviral vectors generated by VectorBuilder expressing full-length mouse *DDX3X* or *DDX3Y*. Viral constructs carried either control mScarlet, mScarlet-tagged DDX3X, or mScarlet-tagged DDX3Y. Culture medium was replaced with fresh medium 24 h after infection, and cells were collected the following day for the experiments.

To test the specificity of antibodies against DDX3X and determine whether they recognize DDX3Y, we used cultured male mouse brain endothelial cells. Brain cortices from aged male mice (22 months) were dissected under sterile conditions on ice. Tissue processing and dissociation were performed using the Adult Brain Dissociation Kit (Miltenyi Biotec, #130-107-677) according to the manufacturer’s instructions. Primary brain endothelial cells were cultured in mouse endothelial cell medium (Cell Biologics, M1168) and used at passage 3. Brain endothelial cells were infected with lentiviral vectors carrying either control mScarlet or mScarlet-tagged DDX3Y. Culture medium was replaced 24 h after infection, and cells were fixed the following day. Cells were then immunostained with an antibody against DDX3X and analyzed.

### Mass spectrometry

Three replicates per condition of rat neuronal culture samples were lysed with 50 mM ammonium bicarbonate and 1 mM calcium chloride with sonication (3 min, 30 Amp, 30-s pulse, a 1-min rest at 4 °C). The protein concentration of the lysate was measured using the Bradford method, and 25 µg of lysate were digested with 0.5 µg of trypsin for 12 h at 37 °C. The concentration of tryptic peptides was measured using the Pierce Quantitative Peptide Assays kit (ThermoFisher Scientific cat# 23275), and 25 µg of peptides were separated on a home-made reverse-phase C18 column in a pipette tip. Peptides were eluted and separated into fifteen fractions using a stepwise gradient of increasing acetonitrile (2%, 4%, 6%, 8%, 10%, 12%, 14%, 16%, 18%, 20%, 22%, 24%, 26%, 28%, 35% acetonitrile) at pH 10. Subsequently, these fractions were combined into five groups (2 + 12 + 22, 4 + 14 + 24, 6 + 16 + 26, 8 + 18 + 28, 10 + 20 + 35) and vacuum-dried. The dried peptide samples were resuspended in a solution of 5% methanol and 0.1% formic acid in water, then analyzed using Orbitrap Fusion mass spectrometers (Thermo Fisher Scientific) coupled with an Easy-nLC 1000 nanoflow LC system (Thermo Fisher Scientific). The analysis utilized an in-house trap column packed with 1.9 μm Reprosil-Pur Basic C18 beads (2 cm × 100 μm) and a 5 cm × 150 μm capillary separation column packed with 1.9 μm Reprosil-Pur Basic C18 beads. The separation was carried out using a 75-min discontinuous gradient of 4–26% acetonitrile and 0.1% formic acid at a flow rate of 800 nL/min. The instrument was operated under the control of Xcalibur software version 4.1 (Thermo Fisher Scientific) in data-dependent mode, acquiring fragmentation spectra of the top 3 seconds. Parent mass spectrum was acquired in the Orbitrap with a full MS range of 300–1400 m/z at the resolution of 120,000. A higher-energy collisional dissociation (HCD) fragmented MS/MS spectrum was acquired in an ion-trap with rapid scan mode. The MS/MS spectra were searched against the target-decoy mouse RefSeq database (release March 23, 2020, containing 62,232 entries) in Proteome Discoverer 2.1 interface (Thermo Fisher) with the Mascot algorithm (Mascot 2.4, Matrix Science). The precursor mass tolerance of 20 ppm and fragment mass tolerance of 0.5 Da were allowed. Two maximum missed cleavages, and dynamic modifications of acetylation of N-term and oxidation of methionine were allowed. Assigned peptides were filtered with a 1% false discovery rate (FDR) using Percolator validation based on q-value. The Peptide Spectrum Matches (PSMs) output from PD2.1 was used to group peptides onto gene level using “gpGrouper” algorithm [101]. An in-housed program, gpGrouper, uses a universal peptide grouping logic to accurately allocate and provide MS1-based quantification across multiple gene products. Gene-protein products (GPs) were quantified using the label-free, intensity-based absolute quantification (iBAQ) approach and normalized to FOT (a fraction of the total protein iBAQ amount per experiment). FOT was defined as an individual protein’s iBAQ divided by the total iBAQ of all identified proteins within one experiment.

### Sample preparation, library preparation, and sequencing

Five replicates per condition of RNA were isolated from primary mouse neurons from both male and female cells using Trizol reagent (Thermo Fisher Scientific, #15596018) according to the manufacturer’s protocol. Sample sizes were based on standard field practice [30, 102, 103]. The total RNA quantity and purity were analyzed using the Bioanalyzer 2100 and RNA 6000 Nano LabChip Kit (Agilent, CA, USA, 5067-1511). High-quality RNA samples with RIN number > 7.0 were used to construct the sequencing library. After total RNA was extracted, mRNA was purified from total RNA (5 μg) using Dynabeads Oligo (dT) (Thermo Fisher Scientific) with two rounds of purification. Following purification, the mRNA was fragmented into short fragments using divalent cations under elevated temperature (Magnesium RNA Fragmentation Module (NEB, #e6150) under 94 °C, 5–7 min). Then the cleaved RNA fragments were reverse-transcribed to create the cDNA by SuperScript™ II Reverse Transcriptase (Invitrogen, #1896649), which were next used to synthesize U-labeled second-stranded DNAs with E. coli DNA polymerase I (NEB, cat.m0209, USA), RNase H (NEB, cat.m0297, USA), and dUTP Solution (Thermo Fisher Scientific, #R0133). An A-base was then added to the blunt ends of each strand, preparing them for ligation to the indexed adapters. Each adapter contained a T-overhang for ligating to the A-tailed fragmented DNA. Dual-index adapters were ligated to the fragments, and size selection was performed with AMPureXP beads. After the heat-labile UDG enzyme (NEB, cat.m0280, USA) treatment of the U-labeled second-stranded DNAs, the ligated products were amplified with PCR by the following conditions: initial denaturation at 95 °C for 3 min; 8 cycles of denaturation at 98 °C for 15 s, annealing at 60 °C for 15 s, and extension at 72 °C for 30 s; and then final extension at 72 °C for 5 min. The average insert size for the final cDNA libraries was 300 ± 50 bp. At last, we performed 2×150 bp paired-end sequencing (PE150) on an Illumina Novaseq™ 6000 according to the vendor’s recommended protocol.

### RNAseq data analysis

A cDNA library constructed by technology from the pooled RNA from primary mouse neuron samples was sequenced using the Illumina Novaseq TM 6000 sequence platform. Using the Illumina paired-end RNA-seq approach, we sequenced the transcriptome, generating 2 × 150 bp paired-end reads. Reads obtained from the sequencing machines include raw reads containing adapters or low-quality bases, which will affect the following assembly and analysis. Thus, to get high-quality, clean reads, reads were further filtered by Cutadapt (https://cutadapt.readthedocs.io/en/stable/,version:cutadapt-1.9). The parameters were as follows: (1) removing reads containing adapters; (2) removing reads containing polyA and polyG; (3) removing reads containing more than 5% of unknown nucleotides (N); (4) removing low-quality reads containing more than 20% of low-quality (Q-value ≤ 20) bases. Then, the sequence quality was verified using FastQC (http://www.bioinformatics.babraham.ac.uk/projects/fastqc/, 0.11.9), including the Q20, Q30, and GC-content of the clean data. We aligned reads of all samples to the mouse reference genome using HISAT2 (https://daehwankimlab.github.io/hisat2/,version:hisat2-2.0.4) package, which initially removes a portion of the reads based on quality information accompanying each read and then maps the reads to the reference genome. HISAT2 allows multiple alignments per read (up to 20 by default) and a maximum of two mismatches when mapping the reads to the reference. HISAT2 builds a database of potential splice junctions and confirms these by comparing the previously unmapped reads against the database of putative junctions. The mapped reads of each sample were assembled using StringTie (http://ccb.jhu.edu/software/stringtie/,version:stringtie-1.3.4d) with default parameters. Then, all transcriptomes from all samples were merged to reconstruct a comprehensive transcriptome using gffcompare software (http://ccb.jhu.edu/software/stringtie/gffcompare.shtml,version:gffcompare-0.9.8). After the final transcriptome was generated, StringTie and ballgown (http://www.bioconductor.org/packages/release/bioc/html/ballgown.html) were used to estimate the expression levels of all transcripts and perform expression abundance for mRNAs by calculating FPKM (fragments per kilobase of transcript per million mapped reads) value. The differential expression analysis was conducted on the quantified expression levels using the “Empirical analysis of DGE” algorithm of the CLC Genomics Workbench 21.0.4 with default settings. This module consists of an implementation of the “Exact Test” for two-group comparisons [104] and incorporated into the EdgeR Bioconductor package [105]. The differentially expressed genes were visualized using a Volcano plot (Advaita Bioinformatics).

### Functional enrichment analysis of RNA-seq data

Differentially expressed genes identified from RNA-seq were analyzed using iPathwayGuide (Advaita Bioinformatics, Ann Arbor, Michigan). The analysis was performed in the context of pathways from the Kyoto Encyclopedia of Genes and Genomes (KEGG) database and gene ontology terms from the Gene Ontology Consortium database [59–61]. The analysis used a probabilistic Over-Representation Analysis (pORA) that incorporates pathway topology information. Enrichment (pORA) and pathway perturbation (pAcc) scores were combined into an overall pathway score using Fisher’s method to calculate a single *p* value. This combined *p*-value was then adjusted for multiple comparisons using both False Discovery Rate (FDR) correction [106, 107] and the more conservative Bonferroni correction [108] to control for Type I errors. These procedures allowed robust identification of pathways significantly affected by the experimental conditions.

### Immunocytochemistry

Cultured primary rat neurons were washed with PBS and fixed with 4% paraformaldehyde-PBS solution for 15 min at room temperature. Cells were rinsed and incubated in 0.1% Triton X-100 in PBS for 5 min at room temperature to permeabilize membranes. Cells were then extensively rinsed with PBS and incubated with 1% bovine serum albumin in PBS for 1 h at room temperature to prevent nonspecific antibody binding. Cells were incubated with a primary antibody solution overnight at 4 °C, washed, and then incubated with a secondary antibody solution for one hour at room temperature. Some neuronal cultures were stained with primary antibodies and with the G4-selective fluorophore N-TASQ overnight at 4 °C. Cells were incubated with secondary antibodies, stained with the 1 µg/mL DAPI dye, and imaged with the Leica DM8i SPE confocal microscope.

### Immunohistochemistry

Human hippocampal samples were obtained from the Histology core at The BRAINS Research Laboratory, Department of Neurology, the University of Texas McGovern Medical School, Houston, TX, under IRB approval HSC-MS-22-0982. Brain samples from a total of six young females, seven aged females, and seven females with Alzheimer’s disease were analyzed (Supplementary Table 1). Alzheimer’s disease was determined per NIA’s guidelines for neuropathologic assessment [109]. The investigators were blinded to group allocation. Following deparaffinization, brain samples (5 µm thickness) were incubated in citrate buffer for 2 cycles of 10 min at 99 °C for antigen retrieval. Then, the samples were washed and incubated with 0.2% bovine serum albumin in PBS as a blocking buffer for 2 h at room temperature. After blocking, samples were incubated with antibodies against DDX3X (1:500) and NeuN (1:1000) overnight. Samples were then incubated with fluorescently labeled secondary antibodies for 1 h at room temperature. The samples were washed and mounted using EverBrite TrueBlack Hardset mounting media with DAPI (Biotium, #23018). Samples were imaged with the Leica DM8i SPE confocal microscope or with AX/AX R with NSPARC Nikon Confocal-based Super Resolution Microscope.

### Correlation analysis from immunohistochemistry

Seven female individuals with Alzheimer’s disease were analyzed. Quantitative image analysis was performed using ImageJ/Fiji. For each individual, the DDX3X puncta index, defined as the number of DDX3X-positive foci per cell, was quantified, and total DDX3X levels were measured as mean fluorescence intensity per cell. Aβ, phosphorylated tau (p-Tau), GFAP, and IBA1 signals were quantified as mean fluorescence intensity within defined regions of interest (ROIs) in the CA1 region of the hippocampus. Correlations were assessed between individuals using Pearson correlation coefficients (r).

### Fluorescence microscopy and image analysis

Living or fixed cells in 24-well plates were imaged using the automated EVOS microscopy system (ThermoFisher Scientific). The microscope was set to image 5% of the well center with the 20X objective and the far-red (RFP), GFP, and DAPI filters. Neurons were also imaged with the Leica DMi8 confocal microscope using a 63X ACS APO 63x/1.30 oil lens. Images were analyzed using ImageJ. Colocalization of fluorescent signals was measured with the EzColocalization plugin for the image processing program ImageJ/Fiji.

### qRT-PCR

Total RNA was extracted from primary cultures using the RNeasy Mini kit (QIAGEN, #74104), and then reverse transcribed using iScript Reverse Transcription SuperMix (Bio-Rad, #1708840), according to the manufacturer’s protocol and as described [26, 110]. RT-qPCR was performed using a Bio-Rad CFX96 Touch machine using SSoAdvanced Universal SYBR Green (Bio-Rad, #1725275) for visualization and quantification according to the manufacturer’s instructions. Primer sequences are listed in Supplementary Table 2. The PCR conditions were 95 °C for 3 min, followed by 40 cycles of 95 °C for 10 s and 55 °C for 30 s. Relative expression levels were calculated from the average threshold cycle number using the delta-delta Ct method.

### RNAscope in situ hybridization

RNAscope in situ hybridization was performed using the RNAscope™ Multiplex Fluorescent Reagent Kit v2 (Advanced Cell Diagnostics, #323100) with minor adaptations. To detect mRNA molecules, RNAscope was performed on FFPE-fixed human brain hippocampal samples. 5 μm sections were cut from FFPE blocks. Briefly, FFPE-fixed section slides were heated at 60 °C for 1 hour before being deparaffinized in xylene and 100% ethanol. After drying the slides for 5 min at RT, H_2_O_2_ was added for 10 min at room temperature. For antigen accessibility, slides were incubated in boiling antigen retrieval solution (99 °C) for 30 min, washed twice with water, dehydrated in 100% ethanol, and finally treated with Protease Plus for 40 min at 40 °C. FFPE-fixed sections were washed twice in PBS. DDX3Y-C1, DDX3X-C2, and NeuN-C3 probes were diluted 1:50 and incubated on slides for 2 h at 40 °C. DDX3Y-C1 probes were detected with TSA-Vivid 650 (1:3000) (Bio-techne, #7527), DDX3X-C2 probes were detected with Opal 570 dye (1:1500) (Akoya biosciences, #FP1488001KT), and NeuN-C3 probes were detected with Opal 520 dye (1:1500) (Akoya biosciences, # FP1487001KT). To quench the autofluorescence due to the accumulation of lipofuscin or other protein aggregates, slides were incubated with autofluorescence eliminator reagent (EMD Millipore, #2160) for 30 s at RT. Slides were mounted using EverBrite TrueBlack Hardset mounting media with DAPI (Biotium, #23018). A one-day protocol has been used in all experiments to maintain slice quality. Samples were imaged with AX/AX R with NSPARC Nikon Confocal-based Super Resolution Microscope.

### Statistical analysis

Statistical analyses were performed using ImageJ and GraphPad Prism. Comparisons between two groups were evaluated using Student’s *t* tests. For experiments involving more than two groups, ANOVA followed by appropriate multiple-comparisons tests was used. A *p-*value < 0.05 was considered statistically significant.

## Supplementary information

Supplemental Legends

Supplemental Table 1

Supplemental Table 2

Supplemental Figure 1

Supplemental Figure 2

Supplemental Figure 3

Supplemental Figure 4

Supplemental Figure 5

Supplemental Figure 6

Supplemental Figure 7

Supplemental Figure 8

Supplemental Figure 9

Supplemental Figure 10

Supplemental Figure 11

Supplemental Figure 12

Supplemental Figure 13

Supplemental Figure 14

Supplemental Figure 15

Supplemental Figure 16

Supplemental Data 1

Supplemental Data 2

Supplemental Data 3

Supplemental Data 4

## Data Availability

All data generated or analyzed during this study are included in its supplementary information files.

## References

[1] Spiegel J, Adhikari S, Balasubramanian S. The structure and function of DNA G-quadruplexes. Trends Chem. 2020;2:123–36. 32923997 10.1016/j.trechm.2019.07.002 PMC7472594

[2] Lyu K, Chow EY, Mou X, Chan TF, Kwok CK. RNA G-quadruplexes (rG4s): genomics and biological functions. Nucleic Acids Res. 2021;49:5426–50. 33772593 10.1093/nar/gkab187 PMC8191793

[3] Rhodes D, Lipps HJ. G-quadruplexes and their regulatory roles in biology. Nucleic Acids Res. 2015;43:8627–37. 26350216 10.1093/nar/gkv862 PMC4605312

[4] Maizels N, Gray LT. The G4 genome. PLoS Genet. 2013;9:e1003468. 23637633 10.1371/journal.pgen.1003468 PMC3630100

[5] Kharel P, Ivanov P. RNA G-quadruplexes and stress: emerging mechanisms and functions. Trends Cell Biol. 2024;771–84. 10.1016/j.tcb.2024.01.005 PMC12069074 38341346

[6] Fay MM, Lyons SM, Ivanov P. RNA G-quadruplexes in biology: principles and molecular mechanisms. J Mol Biol. 2017;429:2127–47. 28554731 10.1016/j.jmb.2017.05.017 PMC5603239

[7] Hansel-Hertsch R, Di Antonio M, Balasubramanian S. DNA G-quadruplexes in the human genome: detection, functions and therapeutic potential. Nat Rev Mol Cell Biol. 2017;18:279–84. 28225080 10.1038/nrm.2017.3

[8] Nurk S, et al. The complete sequence of a human genome. Science. 2022;376:44–53. 35357919 10.1126/science.abj6987 PMC9186530

[9] Smeds L. et al. Non-canonical DNA in human and other ape telomere-to-telomere genomes. Nucleic Acids Res. 2025;53:gkaf298. 40226919 10.1093/nar/gkaf298 PMC11995269

[10] Marsico G, et al. Whole-genome experimental maps of DNA G-quadruplexes in multiple species. Nucleic Acids Res. 2019;47:3862–74. 30892612 10.1093/nar/gkz179 PMC6486626

[11] Besnard E, et al. Unraveling cell type-specific and reprogrammable human replication origin signatures associated with G-quadruplex consensus motifs. Nat Struct Mol Biol. 2012;19:837–44. 22751019 10.1038/nsmb.2339

[12] Damas J, et al. Mitochondrial DNA deletions are associated with non-B DNA conformations. Nucleic Acids Res. 2012;40:7606–21. 22661583 10.1093/nar/gks500 PMC3439893

[13] Spiegel J, et al. G-quadruplexes are transcription factor binding hubs in human chromatin. Genome Biol. 2021;22:117. 33892767 10.1186/s13059-021-02324-z PMC8063395

[14] Chambers VS, et al. High-throughput sequencing of DNA G-quadruplex structures in the human genome. Nat Biotechnol. 2015;33:877–81. 26192317 10.1038/nbt.3295

[15] Esnault C, et al. G4access identifies G-quadruplexes and their associations with open chromatin and imprinting control regions. Nat Genet. 2023;55:1359–69. 37400615 10.1038/s41588-023-01437-4

[16] Robinson J, Raguseo F, Nuccio SP, Liano D, Di Antonio M. DNA G-quadruplex structures: more than simple roadblocks to transcription? Nucleic Acids Res. 2021;49:8419–31. 34255847 10.1093/nar/gkab609 PMC8421137

[17] Wulfridge P, et al. G-quadruplexes associated with R-loops promote CTCF binding. Mol Cell. 2023;83:3064–.e3065. 37552993 10.1016/j.molcel.2023.07.009 PMC10529333

[18] Hansel-Hertsch R, et al. G-quadruplex structures mark human regulatory chromatin. Nat Genet. 2016;48:1267–72. 27618450 10.1038/ng.3662

[19] Biffi G, Tannahill D, Miller J, Howat WJ, Balasubramanian S. Elevated levels of G-quadruplex formation in human stomach and liver cancer tissues. PLOS ONE. 2014;9:e102711. 25033211 10.1371/journal.pone.0102711 PMC4102534

[20] Sauer M, Paeschke K. G-quadruplex unwinding helicases and their function in vivo. Biochem Soc Trans. 2017;45:1173–82. 28939694 10.1042/BST20170097

[21] Lejault P, Mitteaux J, Sperti FR, Monchaud D. How to untie G-quadruplex knots and why?. Cell Chem Biol. 2021;28:436–55. 33596431 10.1016/j.chembiol.2021.01.015

[22] Tabor N, et al. Differential responses of neurons, astrocytes, and microglia to G-quadruplex stabilization. Aging. 2021;13:15917–41. 34139671 10.18632/aging.203222 PMC8266374

[23] Diaz Escarcega R, Marshall P, Tsvetkov AS. G-quadruplex DNA and RNA in cellular senescence. Front Aging. 2024;5:1491389. 39444378 10.3389/fragi.2024.1491389 PMC11496277

[24] Vijay Kumar MJ, Morales R, Tsvetkov AS. G-quadruplexes and associated proteins in aging and Alzheimer’s disease. Front Aging. 2023;4:1164057. 37323535 10.3389/fragi.2023.1164057 PMC10267416

[25] Kallweit L, et al. Chronic RNA G-quadruplex accumulation in aging and Alzheimer’s disease. eLife. 2025;14:RP105446. 39992714 10.7554/eLife.105446 PMC11850002

[26] Moruno-Manchon JF, et al. Small-molecule G-quadruplex stabilizers reveal a novel pathway of autophagy regulation in neurons. eLife. 2020;9:e52283. 32043463 10.7554/eLife.52283 PMC7012600

[27] Rodriguez R, et al. A novel small molecule that alters shelterin integrity and triggers a DNA-damage response at telomeres. J Am Chem Soc. 2008;130:15758–9. 18975896 10.1021/ja805615w PMC2746963

[28] Xu H, Hurley LH. A first-in-class clinical G-quadruplex-targeting drug. The bench-to-bedside translation of the fluoroquinolone QQ58 to CX-5461 (Pidnarulex). Bioorg Med Chem Lett. 2022;77:129016. 36195286 10.1016/j.bmcl.2022.129016

[29] Scheibye-Knudsen M, et al. Cockayne syndrome group A and B proteins converge on transcription-linked resolution of non-B DNA. Proc. Natl. Acad. Sci. U.S.A. 2016;113:12502–7. 27791127 10.1073/pnas.1610198113 PMC5098674

[30] Escarcega RD, et al. Pirh2-dependent DNA damage in neurons induced by the G-quadruplex ligand pyridostatin. J Biol Chem. 2023;229. 10.1016/j.jbc.2023.105157 PMC10534229 37579947

[31] Tabor N, et al. Differential responses of neurons, astrocytes, and microglia to G-quadruplex stabilization. Aging. 2021;13:15917–41. 34139671 10.18632/aging.203222 PMC8266374

[32] Galkin F, et al. Biohorology and biomarkers of aging: Current state-of-the-art, challenges and opportunities. Ageing Res Rev. 2020;60:101050. 32272169 10.1016/j.arr.2020.101050

[33] Seale K, Horvath S, Teschendorff A, Eynon N, Voisin S. Making sense of the ageing methylome. Nat Rev Genet. 2022;23:585–605. 35501397 10.1038/s41576-022-00477-6

[34] Labbadia J, Morimoto RI. The biology of proteostasis in aging and disease. Annu Rev Biochem. 2015;84:435–64. 25784053 10.1146/annurev-biochem-060614-033955 PMC4539002

[35] Hägg S, Jylhävä J. Sex differences in biological aging with a focus on human studies. eLife. 2021;10:e63425. 33982659 10.7554/eLife.63425 PMC8118651

[36] Mauvais-Jarvis F, et al. Sex and gender: modifiers of health, disease, and medicine. Lancet. 2020;396:565–82. 32828189 10.1016/S0140-6736(20)31561-0 PMC7440877

[37] Podcasy JL, Epperson CN. Considering sex and gender in Alzheimer's disease and other dementias. Dialogues Clin Neurosci. 2016;18:437–46. 28179815 10.31887/DCNS.2016.18.4/cepperson PMC5286729

[38] Honarpisheh P, McCullough LD. Sex as a biological variable in the pathology and pharmacology of neurodegenerative and neurovascular diseases. Br J Pharmacol. 2019;176:4173–92. 10.1111/bph.14675 PMC6877795 30950038

[39] Skuse DH. X-linked genes and mental functioning. Hum Mol Genet. 2005;14:R27–32. Spec No 1. 15809269 10.1093/hmg/ddi112

[40] Davis EJ, et al. Sex-specific association of the X chromosome with cognitive change and tau pathology in aging and Alzheimer's disease. JAMA Neurol. 2021;78:1249–54. 34424272 10.1001/jamaneurol.2021.2806 PMC8383157

[41] Davis EJ, et al. A second X chromosome contributes to resilience in a mouse model of Alzheimer’s disease. Sci Transl Med. 2020;12:eaaz5677. 32848093 10.1126/scitranslmed.aaz5677 PMC8409261

[42] Gadek M, et al. Aging activates escape of the silent X chromosome in the female mouse hippocampus. Sci Adv. 2025;11:eads8169. 40043106 10.1126/sciadv.ads8169 PMC11881916

[43] Xu H, et al. CX-5461 is a DNA G-quadruplex stabilizer with selective lethality in BRCA1/2 deficient tumours. Nat Commun. 2017;8:14432. 28211448 10.1038/ncomms14432 PMC5321743

[44] van Schie JJM, et al. Warsaw breakage syndrome-associated DDX11 helicase resolves G-quadruplex structures to support sister chromatid cohesion. Nat Commun. 2020;11:4287. 32855419 10.1038/s41467-020-18066-8 PMC7452896

[45] López CR, et al. Yeast Sub1 and human PC4 are G-quadruplex-binding proteins that suppress genome instability at co-transcriptionally formed G4 DNA. Nucleic Acids Res. 2017;45:5850–62. 28369605 10.1093/nar/gkx201 PMC5449603

[46] Gao J, et al. Yeast transcription co-activator Sub1 and its human homolog PC4 preferentially bind to G-quadruplex DNA. Chem Commun. 2015;51:7242–4. 10.1039/c5cc00742a PMC4465349 25813861

[47] Ishiguro A, Lu J, Ozawa D, Nagai Y, Ishihama A. ALS-linked FUS mutations dysregulate G-quadruplex-dependent liquid-liquid phase separation and liquid-to-solid transition. J Biol Chem. 2021;297:101284. 34624313 10.1016/j.jbc.2021.101284 PMC8567205

[48] Gonzalez V, Guo K, Hurley L, Sun D. Identification and characterization of nucleolin as a c-myc G-quadruplex-binding protein. J Biol Chem. 2009;284:23622–35. 19581307 10.1074/jbc.M109.018028 PMC2749137

[49] Herdy B, et al. Analysis of NRAS RNA G-quadruplex binding proteins reveals DDX3X as a novel interactor of cellular G-quadruplex-containing transcripts. Nucleic Acids Res. 2018;46:11592–604. 30256975 10.1093/nar/gky861 PMC6265444

[50] Chao CH, et al. DDX3, a DEAD box RNA helicase with tumor growth-suppressive property and transcriptional regulation activity of the p21waf1/cip1 promoter, is a candidate tumor suppressor. Cancer Res. 2006;66:6579–88. 16818630 10.1158/0008-5472.CAN-05-2415

[51] Wu DW, et al. DDX3 enhances oncogenic KRAS‑induced tumor invasion in colorectal cancer via the β‑catenin/ZEB1 axis. Oncotarget. 2016;7:22687–99. 27007150 10.18632/oncotarget.8143 PMC5008392

[52] Wu DW, et al. DDX3 loss by p53 inactivation promotes tumor malignancy via the MDM2/Slug/E-cadherin pathway and poor patient outcome in non-small-cell lung cancer. Oncogene. 2014;33:1515–26. 23584477 10.1038/onc.2013.107

[53] Cargill MJ, Morales A, Ravishankar S, Warren EH. RNA helicase, DDX3X, is actively recruited to sites of DNA damage in live cells. DNA Repair (Amst). 2021;103:103137. 34083132 10.1016/j.dnarep.2021.103137 PMC8544569

[54] Dumas L, et al. RNA G-quadruplexes control mitochondria-localized mRNA translation and energy metabolism. Nat Commun. 2025;16:3292. 40195294 10.1038/s41467-025-58118-5 PMC11977240

[55] Mossa A, et al. Sex-specific perturbations of neuronal development caused by mutations in the autism risk gene DDX3X. Nat Commun. 2025;16:4512. 40374608 10.1038/s41467-025-59680-8 PMC12081640

[56] Pickard AJ, Bierbach U. The cell’s nucleolus: an emerging target for chemotherapeutic intervention. ChemMedChem. 2013;8:1441–9. 23881648 10.1002/cmdc.201300262 PMC3893319

[57] Laguerre A, et al. Visualization of RNA-Quadruplexes in Live Cells. J Am Chem Soc. 2015;137:8521–5. 26056849 10.1021/jacs.5b03413

[58] Singh S, Berroyer A, Kim M, Kim N. Yeast nucleolin Nsr1 impedes replication and elevates genome instability at an actively transcribed guanine-rich G4 DNA-forming sequence. Genetics. 2020;216:1023–37. 33106247 10.1534/genetics.120.303736 PMC7768239

[59] Kanehisa M, Goto S. KEGG: kyoto encyclopedia of genes and genomes. Nucleic Acids Res. 2000;28:27–30. 10592173 10.1093/nar/28.1.27 PMC102409

[60] Kanehisa M, Goto S, Kawashima S, Nakaya A. The KEGG databases at GenomeNet. Nucleic Acids Res. 2002;30:42–46. 11752249 10.1093/nar/30.1.42 PMC99091

[61] Ashburner M, et al. Gene ontology: tool for the unification of biology. The Gene Ontology Consortium. Nat Genet. 2000;25:25–29. 10802651 10.1038/75556 PMC3037419

[62] Venkataramanan S, Gadek M, Calviello L, Wilkins K, Floor SN. DDX3X and DDX3Y are redundant in protein synthesis. Rna. 2021;27:1577–88. 34535544 10.1261/rna.078926.121 PMC8594478

[63] Zainu A, et al. FIGNL1-FIRRM is essential for meiotic recombination and prevents DNA damage-independent RAD51 and DMC1 loading. Nat Commun. 2024;15:7015. 39147779 10.1038/s41467-024-51458-8 PMC11327267

[64] Zhou Y, et al. Overexpressed DDX3x promotes abdominal aortic aneurysm formation and activates AKT in ApoE knockout mice. Biochem Biophys Res Commun. 2022;634:138–44. 36242920 10.1016/j.bbrc.2022.09.077

[65] Sapieha P, Mallette FA. Cellular senescence in postmitotic cells: beyond growth arrest. Trends Cell Biol. 2018;28:595–607. 29704982 10.1016/j.tcb.2018.03.003

[66] von Zglinicki T, Wan T, Miwa S. Senescence in post-mitotic cells: a driver of aging? Antioxid Redox Signal. 2021;34:308–23. 32164429 10.1089/ars.2020.8048 PMC7821432

[67] Jurk D, et al. Postmitotic neurons develop a p21-dependent senescence-like phenotype driven by a DNA damage response. Aging Cell. 2012;11:996–1004. 22882466 10.1111/j.1474-9726.2012.00870.x PMC3533793

[68] Ishikawa S, Ishikawa F. Proteostasis failure and cellular senescence in long-term cultured postmitotic rat neurons. Aging Cell. 2020;19:e13071. 31762159 10.1111/acel.13071 PMC6974705

[69] Herdy JR, et al. Increased post-mitotic senescence in aged human neurons is a pathological feature of Alzheimer’s disease. Cell Stem Cell. 2022;29:1637–e1636. 36459967 10.1016/j.stem.2022.11.010 PMC10093780

[70] Sah E, et al. The cellular senescence stress response in post-mitotic brain cells: cell survival at the expense of tissue degeneration. Life. 2021;11:229. 33799628 10.3390/life11030229 PMC7998276

[71] Herdy JR, Mertens J, Gage FH. Neuronal senescence may drive brain aging. Science. 2024;384:1404–6. 38935713 10.1126/science.adi3450 PMC11737875

[72] Funahashi Y, et al. Phosphorylation of Npas4 by MAPK regulates reward-related gene expression and behaviors. Cell Rep. 2019;29:3235–.e3239. 31801086 10.1016/j.celrep.2019.10.116

[73] Korb E, Wilkinson CL, Delgado RN, Lovero KL, Finkbeiner S. Arc in the nucleus regulates PML-dependent GluA1 transcription and homeostatic plasticity. Nat Neurosci. 2013;16:874–83. 23749147 10.1038/nn.3429 PMC3703835

[74] Wainer Katsir K, Linial M. Human genes escaping X-inactivation revealed by single-cell expression data. BMC Genomics. 2019;20:201. 30871455 10.1186/s12864-019-5507-6 PMC6419355

[75] Hoelzl S, Hasenbein TP, Engelhardt S, Andergassen D. Aging promotes reactivation of the Barr body at distal chromosome regions. Nat Aging. 2025;5:984–96. 40312568 10.1038/s43587-025-00856-8 PMC12176624

[76] Kallweit L, et al. Chronic RNA G-quadruplex accumulation in aging and Alzheimer’s disease. Elife. 2025;14:RP105446. 39992714 10.7554/eLife.105446 PMC11850002

[77] Suberbielle E, et al. DNA repair factor BRCA1 depletion occurs in Alzheimer's brains and impairs cognitive function in mice. Nat Commun. 2015;6:8897. 26615780 10.1038/ncomms9897 PMC4674776

[78] Suberbielle E, et al. Physiologic brain activity causes DNA double-strand breaks in neurons, with exacerbation by amyloid-beta. Nat Neurosci. 2013;16:613–21. 23525040 10.1038/nn.3356 PMC3637871

[79] Kim YS, Lee SG, Park SH, Song K. Gene structure of the human DDX3 and chromosome mapping of its related sequences. Mol Cells. 2001;12:209–14. 11710523

[80] Chan CH, Chen CM, Lee YW, You LR. DNA damage, liver injury, and tumorigenesis: consequences of DDX3X loss. Mol Cancer Res. 2019;17:555–66. 30297359 10.1158/1541-7786.MCR-18-0551

[81] Foresta C, Ferlin A, Moro E. Deletion and expression analysis of AZFa genes on the human Y chromosome revealed a major role for DBY in male infertility. Hum Mol Genet. 2000;9:1161–9. 10767340 10.1093/hmg/9.8.1161

[82] Mo J, et al. DDX3X: structure, physiologic functions and cancer. Mol Cancer. 2021;20:38. 33627125 10.1186/s12943-021-01325-7 PMC7903766

[83] Levy T, et al. DDX3X Syndrome: summary of findings and recommendations for evaluation and care. Pediatr Neurol. 2023;138:87–94. 36434914 10.1016/j.pediatrneurol.2022.10.009

[84] Hoye ML, et al. Aberrant cortical development is driven by impaired cell cycle and translational control in a DDX3X syndrome model. Elife. 2022;11:e78203. 35762573 10.7554/eLife.78203 PMC9239684

[85] Shen H, et al. Sexually dimorphic RNA helicases DDX3X and DDX3Y differentially regulate RNA metabolism through phase separation. Mol Cell. 2022;82:2588–.e2589. 35588748 10.1016/j.molcel.2022.04.022 PMC9308757

[86] Sano S, et al. Hematopoietic loss of Y chromosome leads to cardiac fibrosis and heart failure mortality. Science. 2022;377:292–7. 35857592 10.1126/science.abn3100 PMC9437978

[87] Forsberg LA, et al. Mosaic loss of chromosome Y in peripheral blood is associated with shorter survival and a higher risk of cancer. Nat Genet. 2014;46:624–8. 24777449 10.1038/ng.2966 PMC5536222

[88] Vermeulen MC, Pearse R, Young-Pearse T, Mostafavi S. Mosaic loss of Chromosome Y in aged human microglia. Genome Res. 2022;32:1795–807. 36041859 10.1101/gr.276409.121 PMC9712627

[89] Kimura A, et al. Loss of chromosome Y in blood, but not in brain, of suicide completers. PLoS One. 2018;13:e0190667. 29300758 10.1371/journal.pone.0190667 PMC5754120

[90] Maynard, S, Fang, EF, Scheibye-Knudsen, M, Croteau, DL, Bohr, VA. DNA damage, DNA repair, aging, and neurodegeneration. Cold Spring Harb Perspect Med. 2015. 10.1101/cshperspect.a025130 PMC4588127 26385091

[91] Zell J, Rota Sperti F, Britton S, Monchaud D. DNA folds threaten genetic stability and can be leveraged for chemotherapy. RSC Chem Biol. 2021. 10.1039/d0cb00151a PMC8885165 35340894

[92] Feng S, Manley JL. Beyond rRNA: nucleolar transcription generates a complex network of RNAs with multiple roles in maintaining cellular homeostasis. Genes Dev. 2022;36:876–86. 36207140 10.1101/gad.349969.122 PMC9575697

[93] Haeusler RA, Engelke DR. Spatial organization of transcription by RNA polymerase III. Nucleic Acids Res. 2006;34:4826–36. 16971453 10.1093/nar/gkl656 PMC1635290

[94] Grummt I. The nucleolus—guardian of cellular homeostasis and genome integrity. Chromosoma. 2013;122:487–97. 24022641 10.1007/s00412-013-0430-0

[95] Tiku V, et al. Small nucleoli are a cellular hallmark of longevity. Nat Commun. 2017;8:16083. 28853436 10.1038/ncomms16083 PMC5582349

[96] Técher H, Koundrioukoff S, Nicolas A, Debatisse M. The impact of replication stress on replication dynamics and DNA damage in vertebrate cells. Nat Rev Genet. 2017;18:535–50. 28714480 10.1038/nrg.2017.46

[97] Ogawa LM, Baserga SJ. Crosstalk between the nucleolus and the DNA damage response. Mol Biosyst. 2017;13:443–55. 28112326 10.1039/c6mb00740f PMC5340083

[98] Bonda DJ, et al. Pathological implications of cell cycle re-entry in Alzheimer's disease. Expert Rev Mol Med. 2010;12:e19. 20584423 10.1017/S146239941000150X PMC2922901

[99] Laguerre A, Wong JM, Monchaud D. Direct visualization of both DNA and RNA quadruplexes in human cells via an uncommon spectroscopic method. Sci Rep. 2016;6:32141. 27535322 10.1038/srep32141 PMC4989495

[100] Zheng KW, et al. Detection of genomic G-quadruplexes in living cells using a small artificial protein. Nucleic Acids Res. 2020;48:11706–20. 33045726 10.1093/nar/gkaa841 PMC7672459

[101] Saltzman AB, et al. gpGrouper: A Peptide Grouping Algorithm for Gene-Centric Inference and Quantitation of Bottom-Up Proteomics Data. Mol Cell Proteom. 2018;17:2270–83. 10.1074/mcp.TIR118.000850 PMC6210220 30093420

[102] Diaz Escárcega R, et al. Sphingosine kinase 2 regulates protein ubiquitination networks in neurons. Mol Cell Neurosci. 2024;130:103948. 38909878 10.1016/j.mcn.2024.103948 PMC13285857

[103] Vijay Kumar MJ, et al. Small molecule-based regulation of gene expression in human astrocytes switching on and off the G-quadruplex control systems. J Biol Chem. 2024;301:108040. 39615684 10.1016/j.jbc.2024.108040 PMC11750478

[104] Robinson MD, Smyth GK. Small-sample estimation of negative binomial dispersion, with applications to SAGE data. Biostatistics. 2007;9:321–32. 17728317 10.1093/biostatistics/kxm030

[105] Robinson MD, McCarthy DJ, Smyth GK. edgeR: a Bioconductor package for differential expression analysis of digital gene expression data. Bioinformatics. 2009;26:139–40. 19910308 10.1093/bioinformatics/btp616 PMC2796818

[106] Benjamini Y, Yekutieli D. The control of the false discovery rate in multiple testing under dependency. Ann Stat. 2001;29:1165–88. 1124.

[107] Benjamini Y, Hochberg Y. Controlling the false discovery rate: a practical and powerful approach to multiple testing. J R Stat Soc: Ser B. 2018;57:289–300.

[108] Bland JM, Altman DG. Multiple significance tests: the Bonferroni method. BMJ. 1995;310:170. 7833759 10.1136/bmj.310.6973.170 PMC2548561

[109] Hyman BT, et al. National Institute on Aging-Alzheimer’s Association guidelines for the neuropathologic assessment of Alzheimer’s disease. Alzheimer's Dement. 2012;8:1–13. 22265587 10.1016/j.jalz.2011.10.007 PMC3266529

[110] Moruno-Manchon JF, et al. The G-quadruplex DNA stabilizing drug pyridostatin promotes DNA damage and downregulates transcription of Brca1 in neurons. Aging. 2017;9:1957–70. 28904242 10.18632/aging.101282 PMC5636668

