# Supplementary material for: Sex-linked helicases DDX3X and DDX3Y regulate G-quadruplex-associated stress in neurons
Source: Cell Death Dis. 2026 Jun 18;17(1):802. doi: 10.1038/s41419-026-08971-z (PMC13578401; doi:10.1038/s41419-026-08971-z)
Supplement: Supplementary file 1 [file 41419_2026_8971_MOESM2_ESM.pdf]

Group	Age	Race	Neuropathology report
AD females	64	Caucasian	A3B2C3
	73	Hispanic	A3B2C3
	76	Hispanic	A3B3C3 + CAA
	81	Caucasian	A3B2C3
	82	AA	A3B3C3 + CAA
	84	Hispanic	A3B2C3 + CAA
	86	AA	A3B3C3 + CAA
Non-AD females	16	Hispanic	no neurodegenerative pathologies
	31	AA	no neurodegenerative pathologies
	32	Hispanic	no neurodegenerative pathologies
	33	AA	no neurodegenerative pathologies
	35	AA	no neurodegenerative pathologies
	38	AA	no neurodegenerative pathologies
	55	AA	PART
	68	Caucasian	no neurodegenerative pathologies
	71	AA	age-related changes
	73	AA	PART
	74	Asian	age-related changes
	77	Caucasian	age-related changes
	80	Hispanic	age-related changes

Neuropathological diagnoses for Alzheimer's disease (AD), the ABC score that include abeta plaque score (A), neurofibrillary tangle score (B) and neuritic plaque score (C), cerebral amyloid angiopathy (CAA) and Lewy body disease (LBD) were determined according to the National Institute on Aging–Alzheimer's Association guidelines for the neuropathologic assessment of Alzheimer's disease[1]. Limbic-predominant age-related TDP-43 encephalopathy neuropathologic changes (LATE-NC) were assessed based on the 2019 international consensus report[2], and primary age-related tauopathy (PART) was diagnosed according to the 2014 consensus criteria[3].

1. Hyman, B.T. *et al.* National Institute on Aging-Alzheimer's Association guidelines for the neuropathologic assessment of Alzheimer's disease. *Alzheimers Dement* **8**, 1-13 (2012).
2. Nelson, P.T. *et al.* Limbic-predominant age-related TDP-43 encephalopathy (LATE): consensus working group report. *Brain* **142**, 1503-1527 (2019).
3. Crary, J.F. *et al.* Primary age-related tauopathy (PART): a common pathology associated with human aging. *Acta Neuropathol* **128**, 755-766 (2014).
