# Supplementary material for: Sex-linked helicases DDX3X and DDX3Y regulate G-quadruplex-associated stress in neurons
Source: Cell Death Dis. 2026 Jun 18;17(1):802. doi: 10.1038/s41419-026-08971-z (PMC13578401; doi:10.1038/s41419-026-08971-z)
Supplement: Supplementary file 2 [file 41419_2026_8971_MOESM1_ESM.docx]

**Supplemental figure legends**

**Supplemental Figure 1. Quarfloxin downregulates multiple proteins in cultured primary neurons**. A total of 351 proteins were downregulated out of 415 proteins altered by quarfloxin treatment. The threshold for expression change was 0.6, and the significance threshold was 0.05.

**Supplemental Figure 2. Biological processes affected by quarfloxin in cultured primary neurons**. A total of 464 Gene Ontology terms were enriched in neurons treated with 2 μM quarfloxin overnight. Affected processes include neuronal projection development (GO:0070050), neuronal differentiation (GO:0045664 and GO:0050767), memory (GO:0007611), central nervous system development (GO:0021954), transcription initiation by RNA polymerase II (GO:0060260), translation (GO:0006412), and RNA localization (GO:0051236).

**Supplemental Figure 3. PC4 is a nuclear protein enriched in the nucleolus**. **(a)** Primary cortical rat neurons were transfected with PC4-BFP and mApple and imaged. PC4-BFP-positive puncta are visible in the neuronal nucleus. Scale bar, 5 μm. **(b)** Primary cortical neurons were transfected with PC4-BFP, fixed, and stained with antibodies against MAP2c and the nuclear dye SYTOX. PC4-BFP-positive puncta are visible in the nucleus. Scale bar, 5 μm. **(c)** Primary cortical neurons were transfected with PC4-mScarlet, fixed, and stained with antibodies against MAP2c and nucleolin and with DAPI. PC4-mScarlet-positive puncta are visible in the nucleus. Scale bar, 5 μm. **(d)** Quantification of the PC4-BFP puncta index. *p* = 0.0061 (t-test). A total of 120 BFP-transfected neurons and 112 PC4-BFP-transfected neurons were analyzed from four experiments. **(e)** Colocalization of PC4-mScarlet and nucleolin signals measured using the EzColocalization plugin in ImageJ/Fiji. A total of 150 neurons were analyzed from four experiments. *p* = 0.0029 (t-test).

**Supplemental Figure 4. The *DDX3Y* mRNA is expressed in male neurons**. **(a)** Real-time PCR amplification plot showing *Ddx3y* signal in male neurons (Figure 3f). **(b)** Real-time PCR amplification plot for *Ddx3y* in female neurons shows no signal, indicating that *Ddx3y* primers specifically detect *Ddx3y* in male neurons (Figure 3g). **(c)** RNAscope analysis of *DDX3X* and *DDX3Y* expression in male (21, 23, and 40 years) and female (32, 33, and 35 years) human hippocampal autopsy samples. The *DDX3Y* signal is detected only in male samples. Scale bar, 20 μm. **(d)** Quantification of *DDX3Y* signal in hippocampal neurons from male and female samples. Three individuals per group were analyzed. Approximately 250 neurons were analyzed per individual. *p* = 0.0064 (t-test). **(e)** Quantification of the puncta index of *DDX3Y* expression in hippocampal neurons from the same cohort. Three individuals per group were analyzed. Approximately 250 neurons were analyzed per individual. *p* = 0.0001 (t-test).

**Supplemental Figure 5. Viral transgene expression following viral infection.** Primary cortical mouse neurons from male and female embryos were infected with control (mScarlet), DDX3X–mScarlet, or DDX3Y–mScarlet (male neurons only) lentiviral constructs at matched multiplicity of infection (MOI). RT–qPCR quantification of the viral reporter *mScarlet* was performed and normalized to *Gapdh*. n.s., (t-test). Data were pooled from eight independent experiments.

**Supplemental Figure 6. Biological processes affected by DDX3X and DDX3Y in neurons**. **(a)** Examples of biological processes affected by DDX3X in female neurons include nervous system development, response to stress, DNA damage response, behavior, inflammatory response, and DNA geometric changes. **(b)** Biological processes affected by DDX3X in male neurons include nervous system development, stress response, DNA damage response, DNA geometric changes, behavior, and the inflammatory response. **(c)** Biological processes affected by DDX3Y in male neurons include responses to stress, nervous system development, DNA damage response, DNA geometric changes, behavior, and the inflammatory response.

**Supplemental Figure 7. DDX3X and DDX3Y regulate transcription with sex-specific effects**. **(a)** Summary of gene networks uniquely regulated by DDX3X in female neurons (pink), DDX3X in male neurons (yellow), and DDX3Y in male neurons (blue). **(b)** Summary of gene networks shared between DDX3X in female neurons, DDX3X in male neurons, and DDX3Y in male neurons. **(c)** Venn diagrams show the overlap of gene networks among groups.

**Supplemental Figure 8. DDX3X and DDX3Y regulate mRNA splicing in neurons**. DDX3X and DDX3Y regulate alternative splicing events, including alternative 3′ and 5′ splice sites (A3SS and A5SS), mutually exclusive exons (MXE), retained introns (RI), and skipped exons (SE). **(a)** Alternative splicing events in control male versus control female neurons. **(b)** Alternative splicing events in DDX3X-overexpressing male neurons versus control male neurons. **(c)** Alternative splicing events in DDX3X-overexpressing female neurons versus control female neurons. **(d)** Alternative splicing events in DDX3X-overexpressing male neurons versus DDX3X-overexpressing female neurons. **(e)** Alternative splicing events in DDX3X-overexpressing male neurons versus DDX3Y-overexpressing male neurons. **(f)** Alternative splicing events in DDX3Y-overexpressing male neurons versus control male neurons. **(g)** Classification of splicing events analyzed in this study.

**Supplemental Figure 9. DDX3X and DDX3Y upregulate DNA damage response genes in primary neurons**. Primary mouse cortical neurons from female and male embryos were infected with control (mScarlet), DDX3X-mScarlet, or DDX3Y-mScarlet (male neurons only) lentiviral constructs at DIV 14. Expression of *Pif1*, *Neil3*, *Top2a*, *Pole*, and *Brca1* was measured by quantitative RT-PCR and normalized to *Gapdh*. **p* < 0.05, ***p* < 0.01 (t-test). Data were pooled from eight independent experiments.

**Supplemental Figure 10. DDX3X and DDX3Y regulates cell cycle gene expression and neuronal area over time. (a)** Primary cortical neurons from female and male embryos were infected with control (mScarlet), DDX3X-mScarlet, or DDX3Y-mScarlet (male neurons only) lentiviral constructs at DIV14. Expression of *Chek2* was measured by quantitative RT-PCR and normalized to *Gapdh*. **p* < 0.05, ***p* < 0.01, *p* = 0.6127 (DDX3Y in male neurons) (t-test). Data were pooled from eight independent experiments. **(b)** Cultured male and female cortical neurons were infected with control (mScarlet), DDX3X-mScarlet, or DDX3Y-mScarlet (male neurons only) lentiviral constructs. Neuronal area was measured at 24-, 48-, and 60-hours post-infection and normalized to the 24-hour time point. Control neurons (mScarlet-female and mScarlet-male showed an increase in relative neuronal area over time, whereas neurons expressing DDX3X or DDX3Y exhibited a reduction in relative neuronal area. Data are presented separately for female neurons (mScarlet-female, n= 187; mScarlet-DDX3X-female, n= 195) and male neurons (mScarlet-male, n=217; mScarlet-DDX3X-male, n=173; mScarlet-DDX3Y-male, n=193). Quantification was performed using ImageJ/Fiji. *P* < 0.0001. Data were pooled from four independent experiments.

**Supplemental Figure 11. DDX3X and DDX3Y regulate the inflammatory response gene network in male (DDX3Y only) and female neurons. (a)** DDX3X alters inflammatory response genes in female neurons (pink), DDX3X in male neurons (yellow), or DDX3Y in male neurons (blue) (GO:0006954). **(b)** Cultured male and female neurons were infected with control (mScarlet), DDX3X-mScarlet, or DDX3Y-mScarlet (male neurons only) lentiviral constructs at DIV 14. Expression of *Cxcl3* was measured by quantitative RT-PCR and normalized to *Gapdh*. **p* < 0.05, ***p* < 0.01 (t-test). Data were pooled from eight independent experiments.

**Supplemental Figure 12. DDX3X and DDX3Y regulate the cellular senescence gene network in male (DDX3Y only) and female neurons.** DDX3X alters cellular senescence genes in female neurons (pink), DDX3X in male neurons (yellow), or DDX3Y in male neurons (blue) (KEGG: 04218). **(b)** Cultured male and female neurons were infected with control (mScarlet), DDX3X-mScarlet, or DDX3Y-mScarlet (male neurons only) lentiviral constructs at DIV14. Expression of *Foxo1 and Cdkn1a* was measured by quantitative RT-PCR and normalized to *Gapdh*. **p* < 0.05, ***p* < 0.01 (t-test). Data were pooled from eight independent experiments.

**Supplemental Figure 13. DDX3X and DDX3Y regulate the behavior gene network (GO: 0007610) in male (DDX3Y only) and female neurons.** DDX3X alters behavior genes in female neurons (pink), DDX3X in male neurons (yellow), or DDX3Y in male neurons (blue).

**Supplemental Figure 14. RT-PCR validation of selected genes in cultured primary neurons**. Cultured male and female cortical neurons were infected with the control mScarlet, DDX3X-mScarlet, or DDX3Y-mScarlet (only in males) virus. The expression of *Arc* was determined by quantitative RT-PCR relative to *Actin*. **p* < 0.05, ***p* < 0.01 (t-test). Data were pooled from eight independent experiments. These results confirm RNA-seq findings.

**Supplemental Figure 15**. **Specificity analysis of DDX3X and DDX3Y antibodies**. **(a)** Brain endothelial cells from aged male mice were infected with control mScarlet or DDX3Y-mScarlet virus and stained with antibodies against DDX3X and with DAPI. Scale bar, 20 μm. **(b)** Correlation analysis of fluorescence intensities in cells from panel (a). Control cells show no correlation between mScarlet and DDX3X signal. Fifty cells were analyzed (R^2^=0.003). Cells expressing DDX3Y-mScarlet show a strong correlation between mScarlet and DDX3X signal. Fifty cells were analyzed (R^2^=0.868). (**c**) Brain tissue from 22-month-old wild-type female mice stained with antibodies against DDX3Y. Non-specific staining is observed. Scale bar, 50 μm.

**Supplemental Figure 16**. **DDX3X expression in the female human hippocampus and its relationship to** Alzheimer’s disease**-associated pathology**. **(a)** Correlation analyses between DDX3X puncta or DDX3X levels and Aβ in CA1 samples from seven female Alzheimer’s disease individuals. Each dot represents one case. Pearson correlation coefficients (r) are shown. **(b)** Correlation analyses between DDX3X puncta index or DDX3X levels and p-Tau. **(c)** Representative images of hippocampal sections from female Alzheimer’s disease cases stained for DDX3X (green), GFAP (magenta), and IBA1 (red), with DAPI (blue). The merged image shows the spatial relationship between the neuronal DDX3X signal and glial activation. Scale bar, 100 μm. **(d)** Correlation between DDX3X puncta or DDX3X levels and GFAP signal. **(e)** Correlation between DDX3X puncta index or DDX3X levels and IBA1 signal. Each dot represents an individual case. Pearson correlation coefficients (r) are indicated in each plot. > 80 cells were analyzed per case.

**Supplemental table and data**

**Supplemental Table 1**. Demographic and neuropathological characteristics of human hippocampal samples used in this study.

**Supplemental Table 2**. Primers used in this study.

**Supplemental Data 1**. Proteomic analysis of primary cortical neurons treated with vehicle or quarfloxin (2 μM overnight). Three replicates per condition.

**Supplemental Data 2**. RNA-seq alternative splicing analysis of neuronal cultures expressing mScarlet, DDX3X-mScarlet, or DDX3Y-mScarlet. Five replicates per condition.

**Supplemental Data 3**. RNA-seq analysis of neuronal cultures expressing mScarlet, DDX3X-mScarlet, or DDX3Y-mScarlet. Five replicates per condition.

**Supplemental Data 4**. DNA damage response gene network (GO:0006974).
